# Combining geophysical prospection and core drilling: Reconstruction of a Late Bronze Age copper mine at Prigglitz‐Gasteil in the Eastern Alps (Austria)

**DOI:** 10.1002/arp.1872

**Published:** 2022-08-02

**Authors:** Peter Trebsche, Ingrid Schlögel, Adrian Flores‐Orozco

**Affiliations:** ^1^ Institute of Archaeologies University of Innsbruck Innsbruck Austria; ^2^ Applied Geophysics Zentralanstalt für Meteorologie und Geodynamik Vienna Austria; ^3^ Research Division Geophysics, Department of Geodesy and Geoinformation TU Wien Vienna Austria

**Keywords:** core drilling, electrical resistivity tomography, induced polarization tomography, Late Bronze Age, prehistoric mining, seismic refraction tomography

## Abstract

Prehistoric mines are often too large and too deep for conventional archaeological excavations. Non‐destructive and minimally invasive methods of prospection can help to overcome these limits. Our case study of a Late Bronze Age opencast mine (ca. 1050 to 780 BC) shows the potential of geophysical prospection methods combined with core drillings. For the reconstruction of this mine, we combined electrical resistivity and induced polarization (IP) tomography, seismic refraction tomography (SRT) and ground penetrating radar (GPR). The geophysical data were collected based on an orthogonal grid of 10 longitudinal and transverse profiles, laid out over an area of ~330 × 300 m. The profiles allowed a three‐dimensional interpolation of the geological units, the mining dumps, the mining areas and the residual mineralization. Additionally, two deep cores were drilled to ground‐truth the geophysical prospection results. They provided information about the stratification at intersections of the measurement grid, and this proved crucial for validating the interpreted geophysical profiles. Each geophysical method applied provided different information for the reconstruction of the site: the electrical resistivity tomography offered the best clues as to the locations of the geological units and the dumps, the seismic refraction tomography visualized the transition between the dump or backfill layers and the underlying bedrock, and the IP measurements revealed residual mineralization. The georadar measurements, on the other hand, did not contribute to the interpretation owing to the limited depth of penetration. Based on the combination of borehole and geophysical data, it was possible to develop a hypothetical model of an open‐pit mine for copper ore that developed in three phases (mines A–C) during the Late Bronze Age. Without the control provided by the core drillings, one of the mining areas (mine A) could not have been correctly identified in the geophysical prospections.

## 1 INTRODUCTION

### 1.1 Context of the study

Traces of prehistoric mining are often discovered through modern mining and only thus become accessible to research. An example is the world's oldest known salt mine in Hallstatt, Upper Austria (Kern et al., 2009). In many instances, discovery is regrettably accompanied by destruction, as, for example, in the cases of the Copper Age gold mine at Sakdrissi in Georgia (Gambashidze & Stöllner, 2016), the Late Bronze Age gold mine at Ada Tepe in Bulgaria (Alexandrov et al., 2018; Haag et al., 2017) and the prehistoric copper mines in Cyprus (Kassianidou et al., 2021; O'Brien, 2015).

Archaeological excavations at prehistoric mines are extremely challenging and expensive, and this often severely limits the area that can be investigated. An alternative approach is to use geophysical methods to gain information about variations in the subsurface in a non‐invasive manner, permitting the mapping of extensive areas while also reducing time and costs (e.g., Aigner et al., 2021; Bücker et al., 2021; Gallistl et al., 2018; Katona et al., 2021; Patterson et al., 2021). For instance, geomagnetic and georadar surveys have successfully delineated the full extent, number and depth of Neolithic flint‐mining shafts at Arnhofen in southern Germany (Faßbinder, 2003; Leopold & Völkel, 2003, 2004). Electrical resistivity tomography, sensitive to the electronic conduction of metallic minerals (e.g., Bücker et al., 2018; Flores Orozco et al., 2015; Flores‐Orozco et al., 2021), was successfully applied in the prospection of the Alpine Bronze Age mining sites on the Mitterberg in the Austrian province of Salzburg, where the results permitted the interpretation of the superficially visible collapsed shafts (German *Furchenpingen*) of the Main Lode and the Brandergang Lode (Herd, 2006; Herd & Taube, 2011; Stöllner, Breitenlechner, et al., 2011, p. 124; Stöllner et al., 2006, pp. 123–124; see also Thomas, 2018, pp. 398–399). Electrical resistivity measurements were also conducted in the Slovak Ore Mountains to investigate a prehistoric mine at the Piesky site in Špania Dolina (Garner et al., 2014, pp. 73–76; Modarressi‐Tehrani & Garner, 2015, p. 51), and similar investigations have been carried out at the Bronze Age tin mines at the Askaraly I and V sites in Central Asian Kazakhstan, although not yet fully published (Stöllner, Samaschev, et al., 2011, p. 239; Stöllner et al., 2013, pp. 368–370).

If different geophysical methods are combined (see, e.g., Bücker et al., 2021; Sea & Ernenwein, 2020; Steiner et al., 2021), interpretative results can be improved. A combined approach has been used to explore ancient underground aqueducts, such as the Tunnel of Eupalinus on Samos, Greece (Tsokas et al., 2014), and the Triglio aqueduct in Apulia, Italy (Leucci et al., 2016). However, the verification of geophysical measurements at great depths by core drilling remains, so far, a rare exception. The present study is based on geophysical measurements conducted at the Bronze Age mine of Prigglitz‐Gasteil on the eastern edge of the Alps in Lower Austria, using a combination of electrical methods (resistivity and induced polarization) and seismic refraction tomography. The interpretation of the results was verified, for the first time in the context of prehistoric mining, by drilling two deep cores, to depths of 32 and 37 m, respectively.

### 1.2 Research objectives

The main objective of the geophysical prospections was the exact localization of the prehistoric mine workings at Prigglitz‐Gasteil. Although the site is well known as a Late Bronze Age copper production site, no traces of copper extraction are recognizable on the surface. Only indirect evidence of copper mining is available, mostly in the form of massive spoil heaps containing waste from both primary mining and the subsequent processing of the ores. However, no depressions, pits or other structures usually indicative of prehistoric ore mining have been detected to date during site walks or in the digital terrain models. A geophysical survey was therefore planned, aimed at (1) determining the thickness of the dump bodies, (2) locating the associated mine workings, (3) reconstructing the type of mining (surface or underground) and (4) estimating the volume of the mine. It was also hoped that the geophysical results would provide information about the geological setting and any remaining mineralization (ore veins). Locating the latter was particularly important for future sampling for the original ores and for assessing whether prehistoric mining stopped because the deposits were exhausted or for other reasons.

## 2 ARCHAEOLOGICAL AND GEOLOGICAL BACKGROUND

### 2.1 The Bronze Age copper mining site of Prigglitz‐Gasteil

The Prigglitz‐Gasteil ‘Cu I’ site (Neunkirchen district, Lower Austria/A) is an extensive prehistoric mining settlement where chalcopyrite was mined, beneficiated, smelted and processed into bronze products. According to a series of radiocarbon dates and some chronologically significant bronze finds, the period of activity of the site lasted from ca. 1050 to 780 BC; in archaeological terminology, it belongs to the younger phase of the Urnfield Period (Ha B2–3) (Trebsche, 2015). Archaeological excavations took place here in 1956 and 1958 under the direction of archaeologist Franz Hampl and mineralogist Robert J. Mayrhofer (Hampl & Mayrhofer, 1963) and again from 2010 to 2014 under archaeologist Peter Trebsche (2010, 2011, 2012, 2013, 2014a, 2014b, 2014c). The excavations on the excellently preserved settlement terraces, which were established on waste dumps immediately adjacent to the mining operations, provide detailed insights into the animal‐ and plant‐based diet of the inhabitants (Heiss et al., 2021; Trebsche & Pucher, 2013) and the various activities related to metal production (Haubner et al., 2015, 2017, 2019; Mödlinger & Trebsche, 2020, 2021; Mödlinger et al., 2021), thus providing a unique insight into the living and working areas, the division of labour and the organizational structure of a relatively small copper production site on the edge of the Eastern Alps.

**FIGURE 1:**
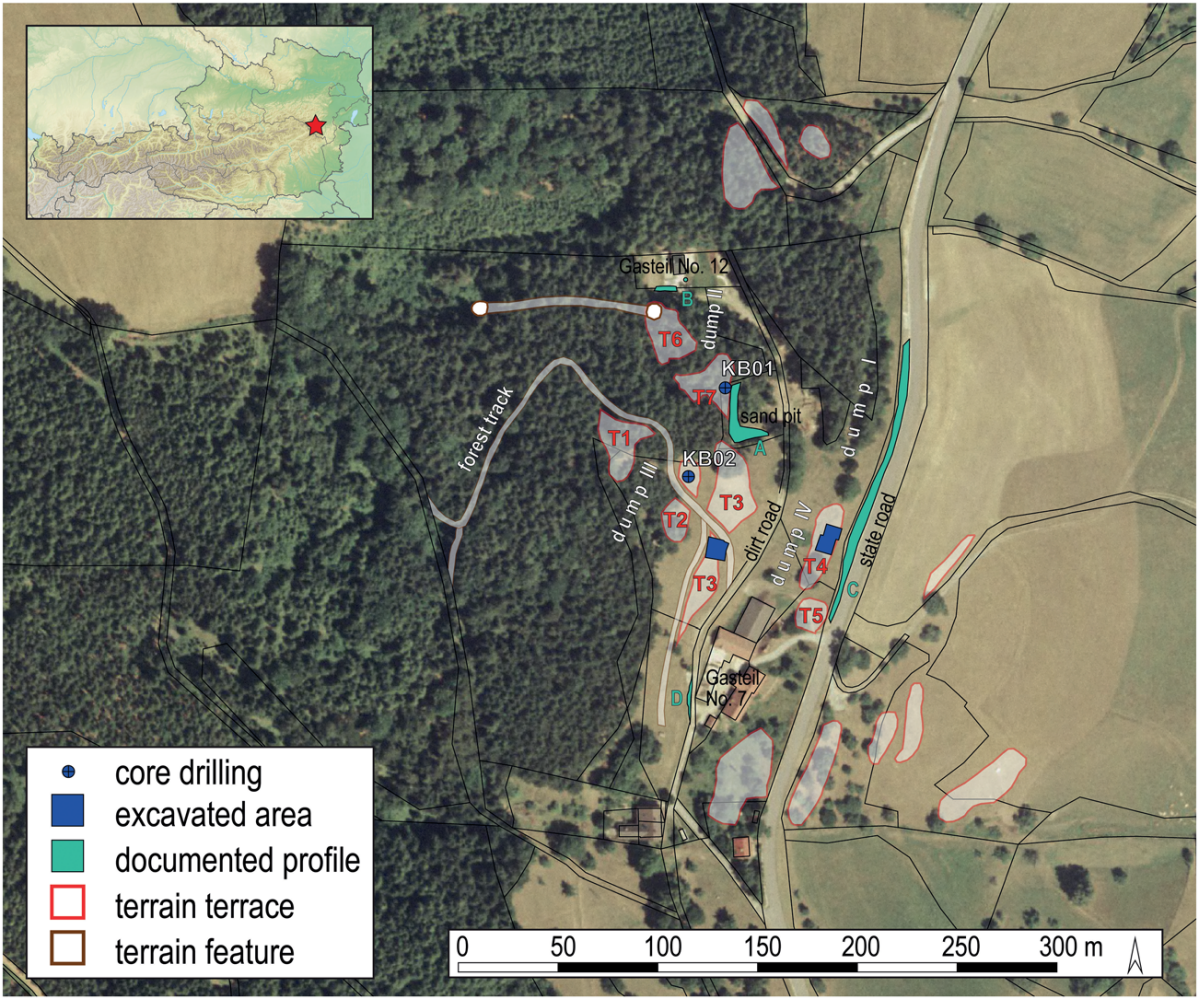
Prigglitz‐Gasteil: (a) location of the study area in Austria, (b) plan of the site showing terrain terraces (T), dumps, excavation areas and core drilling sites (KB) (map of Austria: © BEV, 2021 with kind permission; orthophoto: © Land Niederösterreich with kind permission; additions by Peter Trebsche) [Colour figure can be viewed at wileyonlinelibrary.com]

The site is located on the eastern slope of the Gahns mountain, in the area of Gasteil farmstead No. 7 (Figure 1). Below the timberline, on the surface of meadows now used as cattle pastures, several massive dump bodies are clearly recognizable, sloping from west to east. They are particularly visible in the embankments of the state road, which runs north–south, and the roadway above the farmhouse (for a detailed description see Trebsche & Weixelberger, 2022). In view of the considerable dump volume, it is astonishing that today no corresponding mass deficit, for example, a large hollow or a fall shaft, is recognizable in the terrain.

### 2.2 Results from the core drilling

The first campaign of geophysical measurements was conducted from 16–18 October 2017, followed by the core drilling that took place between 23 October and 9 November 2017. The two drillings were located on the artificial terrain terraces T2 and T7, near intersections of the geophysical survey lines and labelled KB01 and KB02 (KB from the German *Kernbohrung*, core drilling) (Figure 1). The terrain morphology and the results of previous pile core probing had indicated that this was where the greatest thickness of waste rock was likely to be. Drilling at KB01 began from an elevation of 726.25 m above sea level on terrace T7 and reached a depth of 32 m. Drilling at KB02 began from an elevation of 732.16 m a.s.l. on T2, the second uppermost terrain terrace, and reached a depth of 37.5 m. Detailed results from the analysis of the cores have already been reported in Trebsche and Weixelberger (2022). In summary, the two cores (drill diameter 219 mm, core diameter 180 mm) showed an analogous stratigraphy. The surface of the base rock was encountered far below the expected former ground surface (at least 19 m in KB01, 29 m in KB02), indicating the presence of an artificial, man‐made pit, which we interpret as a large opencast copper‐ore mine. The first layer of fill consisted of mining debris of different grain size with a varying degree of metaquarz wacke, limestone, porphyrite and residual iron ore as well as a small proportion of residual copper ore. This was covered by a layer of material from a massive landslide event. On top of this, more mining debris was deposited, until the original mine pit was completely filled in. These processes explain why no traces of the mine workings are now visible in the terrain. In general, the direct analysis of the materials recovered from coring provided the basis for the interpretation of the geophysical imaging results.

### 2.3 Geological background and ore deposits

**FIGURE 2:**
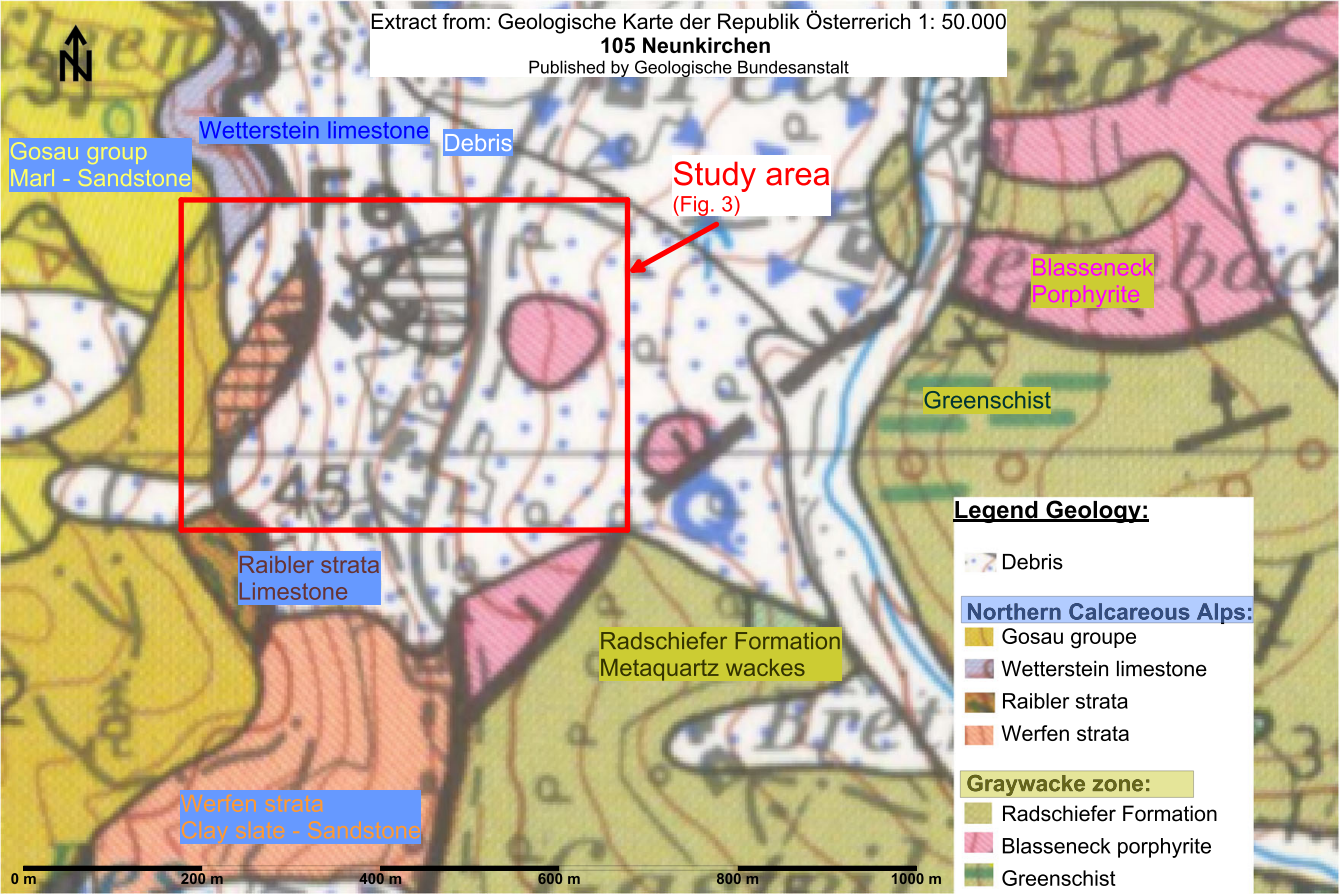
Detail of the Austrian Geological Map, Section 105 (Neunkirchen) (© Geologische Bundesanstalt, with kind permission; additions by Ingrid Schlögel) [Colour figure can be viewed at wileyonlinelibrary.com]

In the Geological Map of the Republic of Austria 1:50 000, sheet 105 Neunkirchen (Herrmann et al., 1992), the former mining area is marked as ‘mining dumps’ (in German ‘Halde’), shown as white areas (Figure 2). The debris covering the slope prevented the geological subsurface from being mapped. Immediately west and south of the study area, rocks belonging to the Northern Limestone Alp formations occur:

- Wetterstein Limestone
- Gosau marl
- Raibl limestones
- Werfen clay slate

These Northern Limestone Alp formations form a sharp tectonic contact (overthrust) with the rocks of the graywacke zone to the east (Noric Nappe, Oberostalpin):

- Metaquartz wackes of the Radschiefer Formation
- Blasseneck porphyrite
- Greenschist

In the study area, therefore, limestones are presumed to occur below the slope debris and anthropogenic dumps in the west, with a sharp transition to metaquartz wackes and porphyrite in the east.

## 3 METHODS

### 3.1 Survey layout

**FIGURE 3:**
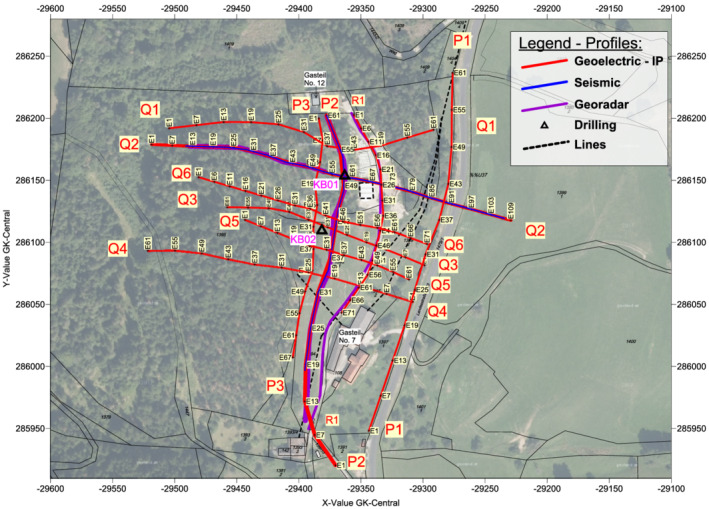
Geophysical measurements conducted at Prigglitz‐Gasteil. Location of the measurement profiles, measurement points, power lines and boreholes [Colour figure can be viewed at wileyonlinelibrary.com]

Before the geophysical measurements began, the heaps were jointly walked by the authors in order to lay the measurement lines across the relevant archaeological terrain features (Figure 3). In principle, the intention was to create an orthogonal grid of survey lines across the mining dumps. The survey lines running approximately west to east, at right‐angles to the contour lines of the site slope, were designated as transverse profiles and labelled with the letter Q, and the measurement points were numbered in the order in which the measurements were taken. The lines running from north to south, approximately parallel to the contour lines of the slope, were designated as longitudinal profiles and labelled with the letters P and R. Due to forestation and minor obstacles, the profiles could not be aligned exactly. In total, an area of approximately 330 × 300 m was prospected. As mentioned above, the core‐drilling sites were placed at or near intersections of the geophysical survey lines. KB01 was located exactly at the intersection of transverse profile Q2 and longitudinal profile P2. KB02 was located midway between the intersections Q3/P2 and Q3/P3.

The first survey took place from 16 to 18 October 2017 and consisted of the collection of six electrical imaging profiles, combining electrical resistivity (ERT) and induced polarization (IP) measurements (P1–P2 and Q1–Q4), two seismic refraction profiles (SRT; P2 and Q2) and three profiles (P2, Q2 and R1) measured with ground penetrating radar (GPR or georadar). These were supplemented by four additional electrical imaging profiles (P3, Q5, Q6 and R1) in a second survey from 2 to 5 July 2018. The profiles were marked out on site with pegs and surveyed with a GPS device (GSM RTK GNSS system from Altus). The positional accuracy recorded and used for data processing was ±2 cm.

### 3.2 Electrical imaging: Electrical resistivity and induced polarization

Both electrical resistivity and induced polarization imaging techniques are based on the injection of current into the ground by means of a pair of electrodes and measuring the resulting voltage across another electrode pair (see Binley & Slater, 2020; Flores Orozco et al., 2018, 2020, 2021). Imaging measurements comprised hundreds of four‐electrode measurements by the combination of dozens of electrodes, to map for lateral and vertical changes in the electrical properties of the subsurface (Flores Orozco et al., 2018, and references therein). Once the current is injected into the ground, most of the free charges (e.g., ions) in the ground (commonly in the pore water) migrate, with the ability of such movement controlled by the electrical conductivity (or its reciprocal the electrical resistivity) of the materials. Moreover, some charges accumulate at the electrical double layer (EDL) formed at the grain‐water interface of the subsurface material, resulting in an energy‐storage or polarization effect. The induced polarization (IP) method measures the electrical impedance, which contains information of both the conductivity and polarization properties, whereas the electrical resistivity tomography (ERT) records only the magnitude of the electrical impedance (i.e., the resistance) and resolves only the resistivity of the subsurface (see, e.g., Binley & Slater, 2020). Inversion of the data sets provide the images of the complex resistivity (i.e., complex conductivity) revealing the spatial variations of the resistivity and polarization properties. Metallic minerals differ from other materials in that they also present free charges that can migrate and polarize within the material during the current injection (see Bücker et al., 2018, 2019, and references therein); thus, resulting in high electrical conductivity r (i.e., lower electrical resistivity) and polarization. Clay materials have a high surface charge; thus, these may also result in a smaller but still measurable polarization response, as well as relatively low electrical resistivity (Bücker et al., 2019, and references therein).

The IP imaging measurements were collected in both time‐domain and frequency‐domain (see Flores Orozco et al., 2020, 2021). For time‐domain measurements, we used a Syscal Pro system (from Iris instruments) with 72 electrodes and 10 measuring channels, while for frequency‐domain measurements, we used a DAS‐1 unit (from Multi Phase Technologies) with 64 electrodes and 8 measuring channels. To avoid contamination of the data due to polarization of the electrodes or electromagnetic coupling, we deployed a dipole–dipole configuration with voltage readings always ahead of the current dipole (Flores Orozco et al., 2021), while the dipole length was defined as three times the electrode spacing. Electrodes were spaced at either 2.5 or 5 m intervals, depending on the accessibility of the terrain (see Table 1). In electrical imaging, the depth of the measurements increases as the distance between the current and potential dipoles increases, while the resolution increases as the distance between the electrodes of each dipole decreases. The measurements planned here aimed at reaching a nominal measurement depth of 40 to 60 m. Imaging geophysical surveys have larger amount of measurements to the middle of the array and only a few readings at the ends; thus, the investigations at deeper at the middle of the imaging plane and lower at the beginning and end of the profile.

**TABLE 1:** Prigglitz‐Gasteil: Geoelectric profiles and their specifications

Name of profile	Electrode arrangement	Electrode distance (m)	Number of electrodes	Length (m)
P1	Dipole–dipole	5	64	314
P2	Dipole–dipole	5	61	297
P3	Dipole–dipole	3	71	210
Q1	Dipole–dipole	4	64	250
Q2	Dipole–dipole	3	112	330
Q3	Dipole–dipole	3	64	187
Q4	Dipole–dipole	4	61	238
Q5	Dipole–dipole	2.5	64	157
Q6	Dipole–dipole	3	71	197
R1	Dipole–dipole	2.5	71	177
Total		—	—	2357

The frequency‐domain data were collected along profiles P1, P2, Q1, Q2, Q3 and Q4 using an acquisition frequency of 1 Hz. The time‐domain measurements were collected along profiles P3, R1, Q5 and Q6, based on 20 measurement windows with a sampling time of 20 ms and a delay of 50 ms after switching the current off, and a pulse length of 500 milliseconds. The acquisition frequency and pulse length were selected to minimize acquisition time (favoured by high frequencies) and avoid contamination of the data by high frequency parasitic electromagnetic fields (Flores Orozco et al., 2021). The transformation of integral chargeability values into apparent phase readings was done using linear approximation, assuming a constant phase angle (i.e., no frequency dependence in the data), as presented in van Voorhis et al. (1973). This approach allowed a quantitative comparison of the imaging results obtained from frequency‐ and time‐domain IP, as demonstrated in Flores Orozco et al. (2012, 2021). Nonetheless different colour maps and scales were used to differentiate between the two IP surveys (values of 1–10 mrads in the frequency‐domain profiles, values of 1–25 mrads in the time‐domain profiles). The higher values observed in the time‐domain measurements, as compared with the frequency‐domain measurements collected at 1 Hz, reflected the frequency dependence in the data, with higher values associated with higher frequencies (i.e., earlier times). To quantify the data error, we used analysis of normal and reciprocal data and of the decay curve, as presented in Flores Orozco et al. (2018). Such analysis results in the quantification of 5% relative error and 0.001 Ω in the absolute error, for the transfer resistances, whereas an error or two miliradians (mrads) for the phase. For the inversion of the IP data, we used a smoothness constrained algorithm (Kemna, 2000), where frequency‐domain readings, at a given frequency, are used to model the distribution of the complex electrical resistivity (i.e., resistivity and polarization) across an imaging plane. In CRTomo, the inversion stops once that the forward solution of the inverted model fits the data to the confidence interval defined by the error model. For further references about the inversion scheme can be found elsewhere (Flores Orozco et al., 2012, 2018; Kemna, 2000).

### 3.3 Seismic refraction tomography

To sustain the interpretation of the electrical data, seismic refraction measurements were made along profiles Q2 and P2 (Table 2). However, due to limited cable length, the seismic profiles were slightly shorter than the electrical profiles. To collect the seismic data, we used 30 Hz vertical geophones firmly placed into the ground as receivers, while seismic waves were generated with hammer blows using an 8 kg sledgehammer. Shot points were made every fourth geophone with a stacking of two hammer blows per shot point; in noisy readings, we used a stacking of four hammer blows. A Geometrics system was used to record the data. The seismic signals were recorded for 512 ms from the start time and at a sampling rate of 0.5 ms.

**TABLE 2:** Prigglitz‐Gasteil: Seismic profiles and their specifications

Name of profile	Geophone distance (m)	Hammerblow distance (m)	Number of geophones	Length (m)	Start/end electrode
P2	3	12	72	213	E18‐E61
Q2	3	12	107	314	E6‐E112
Total	—	—	—	527	—

Travel time analysis was conducted to calculate the depth of the refraction interface and its associated seismic velocity using the ProMAX® industrial processing system (Landmark Graphics Corp.). Following the picking of the first arrivals, the resulting travel times were inverted using the Rayfract package to resolve for the variations in the seismic velocities. The Rayfract software is based on the wavepath eikonal traveltime (WET) inversion algorithms. Hence, the wavepaths are modelled in actual 2D geometries, which take into account topography changes, For further reference on the inversion algorithm, we recommend Schuster and Quintus‐Bosz (1993). We present inversion results obtained after 20 iterations, which resulted in a root‐mean‐square (RMS) error of 5.9% and 3.2% for data collected along Q2 and P2, respectively. Such discrepancy between the data and the forward response of the modelled data is quite low and evidences the good data quality and the adequate identification of the first arrivals. For the inversions, we used a start model defined by a gradient function (the seismic velocities increasing a depth), with the values automatically selected by the software.

The depth of penetration in seismic refraction tomography depends on the one hand on the profile length, frequency and strength of the seismic source and the seismic velocities in the subsurface on the other hand. With the profile lengths available here, the use of a sledge hammer as the source, and the relatively high velocities in the subsurface, a penetration depth of more than 60 m could be achieved in the central areas of the profiles, reducing to about 20 m at the ends.

### 3.4 Ground penetrating radar

Three lines of ground penetrating radar data were measured using a MALÅ GroundExplorer (GX) device and 80 and 160 MHz antennas. Profile R1 was located on the wide driveway leading to the modern timber storage area. The second line was measured along profile P2, and the third line overlapped with part of profile Q2, from profile metre 30 to profile metre 150. The individual antenna profiles were height corrected, processed as vertical 2D sections using the program ReflexW and displayed using the Surfer11 modelling tool (GoldenSoftware). GPR measurements were taken from 16 to 18 October 2017 during a dry weather period.

### 3.5 Data combination and volume calculations

Each electrical imaging profile was interpreted in relation to geological units, dumps and mine workings, incorporating seismic data where available. The boundaries of the interpreted geological units were inferred by linear interpolation, their geometry was interpolated using common kriging and the results transferred to the site plan using the Surfer program from Golden Software LCC.

For the calculation of the volume of the different units, the depths of the interpreted interfaces were digitized at every third/fourth electrode position. After that, the calculation of the thicknesses was made in the program Microsoft EXCEL. The actual surface of the terrain was used as the upper boundary for calculating the volume of the dumps. The upper limit of the mining areas was either the upper edge of the terrain or the lower edge of the dump. The landslip material in mining area A could not be considered in the volume calculation because it could not be clearly delineated in the geophysical profiling.

## 4 RESULTS AND INTERPRETATION

### 4.1 Transverse profile Q2

**FIGURE 4:**
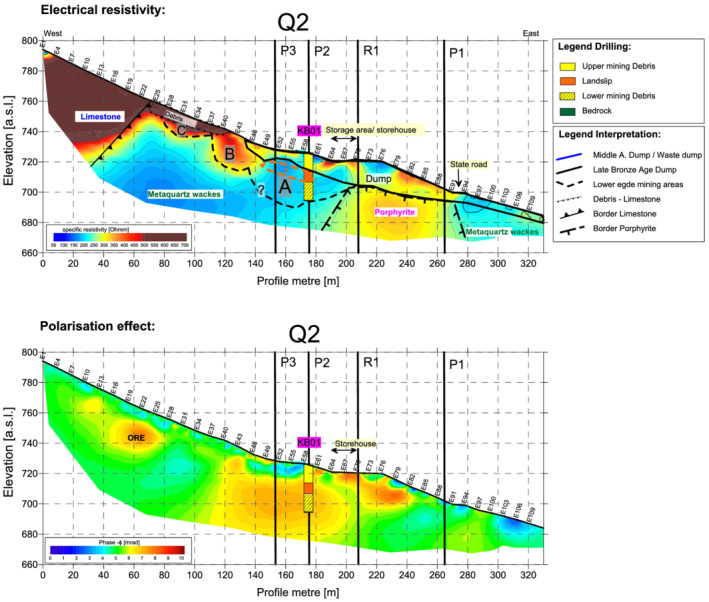
Prigglitz‐Gasteil: Profile Q2: (a) resistivity and (b) induced polarization imaging results [Colour figure can be viewed at wileyonlinelibrary.com]

**FIGURE 5:**
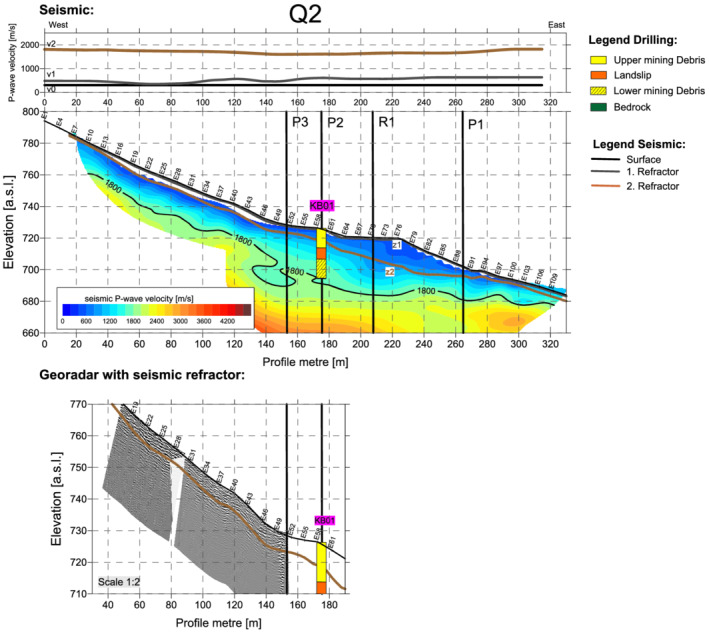
Prigglitz‐Gasteil. Profile Q2: (a) refraction seismic and refraction tomography and (b) georadar [Colour figure can be viewed at wileyonlinelibrary.com]

The key to the interpretation was provided by transverse profile Q2, which traversed the entire site from west to east, that is, a distance of 330 m, and for which a control was provided by the KB01 core. In terms of terrain morphology, profile Q2 (Figures 4 and 5) began with a section of natural slope (profile metres 0–120), which steepened and then levelled off at the horizontal dump surface of artificial terrace 7 (profile metres 155–175). This horizontal section then gave way to a steep artificial slope created by sand mining which began in the 1950s, terminating in a levelled timber yard with a storehouse (profile metres 190–225). East of this, the dump continued, interrupted only by the modern cutting for the state road (profile metres 270–280). Core drill hole KB01 was located at profile metre 174 (electrode point 59, abbreviated: E59).

The resistivity model for the Q2 profile (Figure 4a) showed clearly contrasting values corresponding to the different geological units. The highest electrical resistivity values (over 500 ohmm), from E1 to E25 in the west, were assigned to the limestones. Based on the results from both drilling cores, the low resistivity values in the east (~200 ohmm) were assigned to the metaquartz wackes, where ore mineralization was also located (Trebsche & Weixelberger, 2022).

The areas with medium resistivity values (about 400 ohmm, profile metres 200–260) were interpreted as porphyrites. Further east, lower resistivity values (250 ohmm) were interpreted as representing members of the Radschiefer formation. This formation also belongs to the graywacke zone but had been separated from the metaquartz wackes by sedimentary or alteration processes. Between E25 and E45, the metaquartz wackes were overlaid by a 10–15 m‐thick layer of slope debris consisting of calcareous alpine material, indicated by very high resistivity values.

The Bronze Age dump body extended from E45 to the end of the profile at E112. The thickness of the dump was about 10 m around E64, increasing to over 17 m in the area of the present timber stockpile. Below the state road (E 94), it decreased to 3 m. The electrical resistivity varied in response to changes in the concentration of iron minerals and the composition of materials, as expected for mining dumps.

Mining area A, recorded in core KB01 to a depth of 32 m, was delineated according to the results of the geoelectric survey. Its backfilling showed low resistivity values similar to those of the underlying metaquartz wackes. Its lower boundary was not clearly discernible in the resistivity image. The eastern edge of the mine was formed by the outcropping porphyrites. On the slope above mine A, in the area of the metaquartz wackes, two other large, trough‐shaped depressions were rendered visible in the resistivity imaging results by the high resistivity values of the backfill material. From their shape, these anomalies were interpreted as two further mining areas. Upper mining area C (between E27 and E37; profile metres ~80–112) had a horizontal floor and an inclined face on the slope (i.e., east) side, giving it a step shape. Mining area B, directly adjacent but lower (located between E37 and E46; profile metres 112~140), had a trough‐shaped floor and a nearly vertical face on the slope side. Mining area C measured ~32 m from east to west and reached a depth of at least 12 m below the former ground surface and 17 m below the present ground surface. Mining area B had a maximum width of ~25–30 m (the boundary between mining areas A and B was not distinct) and a minimum depth of 23 m below the former ground surface and ~25 m below today's ground surface. Mining area B was first backfilled with material characterized by medium resistivity values and then overlain with uniform material associated with high resistivity values. It was assumed that mine B was stratigraphically older than mine C. It seemed likely that mine B was backfilled with the overburden from the upslope mine C. Mine C was subsequently partially backfilled and the remaining hollow filled by a layer of natural calcareous alpine slope debris, about 8–10 m thick, leaving no trace of the mine visible in the terrain today.

The fill of mining area A between electrodes 46 and 64 showed evidence of an elevated polarization effect (Figure 4b), indicating the presence of metallic ores. This could be explained either by residual copper mineralization or by iron ores in the Bronze Age dump. Bronze Age dumps often contain a high proportion of siderite, an iron oxide that was not usable by Bronze Age miners and was therefore deposited in dumps (Trebsche & Weixelberger, 2022). However, siderite does not usually produce a measurable polarization effect at the frequencies under investigation (Abdel Aal et al., 2014). The high polarization response is therefore more likely to indicate concentrations of copper ore, which commonly increases the polarization response (e.g., Flores‐Orozco et al., 2021) or magnetite, another iron oxide that also enhances polarization (Hubbard et al., 2014). Another area with elevated polarization values was located between E16 and E28 at a depth of about 7–17 m, that is, exactly in the border zone between the limestones and the metaquartz wackes. This could be residual mineralization that was not fully extracted by mining activities in area C. Limestones and porphyrites are generally characterized by low polarization values at the low frequencies studied here (1 Hz).

The seismic analysis (Figure 5a) of profile Q2 revealed a three‐layer refraction model. For each seismic signal, the respective seismic velocities of the three layers (v0, v1 and v2) were compared with the depths of the refraction interfaces (z1 and z2) and the height in m above sea level (a.s.l.) of the receptor. The first layer corresponded to the topsoil. The bottom of this layer (z1) was located at a depth of 0.5–1 m. The second layer corresponded to the slope debris or the waste rock (loose rock). At the beginning of the profile (in the west,) the bottom of the second layer (z2) was located at a depth of about 2–6 m but became deeper around E55, reaching a maximum depth of about 18 m at E77. Thereafter, it ran almost horizontally as far as the state road E91 and rose again to around 3 m at the eastern end of the profile. The contact between the two units (i.e., the refractor) is characterized by the velocity of the lower unit, which in this case was found around 1700 m/s.

In the area of the limestones, extending as far as E45, the refraction interface z2 represented the transition from slope debris to highly weathered limestone. From E45 onwards, comparison with the resistivity and induced polarization models and the drilling cores showed that z2 could be interpreted as the boundary between the bottom of the dump material and the bedrock.

The velocity distribution resolved in the seismic tomogram indicated that the 1800 m/s isoline marked the transition to the solid bedrock (limestones, metaquartz wackes and porphyrites) with a higher increase in seismic velocity after the 1800 m/s isoline. From E46 onwards, the depth of this interface dropped steeply and then remained at about the same level until the end of the profile. Between E46 and E67, the 1800 m/s isoline can therefore be interpreted as the lower limit of mining area A.

The data from the georadar survey (Figure 5b) showed a barely significant penetration depth of only about 5–8 m (depth of 2nd seismic refractor z2), indicating structures near the surface but providing no information about the depth of the mines or mining dumps, as the profile ended also just before the dumping area. It will therefore not be further discussed.

### 4.2 Transverse profile Q1

Transverse profile Q1 (Figure S1) ran from west to east and was the most northerly transverse profile measured. From the start of the profile to electrode 30, it ran along a steep trench clearly visible in the terrain. At electrode 30 there was a possible entrance hole (Hampl & Mayrhofer, 1963, p. 59; Figure 11). From E34 to E41, it traversed the youngest dump, dump II, which probably belonged to a mediaeval or modern exploratory iron‐ore mine (Trebsche & Weixelberger, 2022). From E43 to E46, it crossed the aforementioned modern log storage area, on the slope below which (E46–E52), according to the landowner, is a modern refuse dump. After E52, the profile ran along dump I and ended just before the cutting for the state road at E64.

From the resistivity imaging results (Figure S1a), as in the case of Q2, the transition from the limestones to the metaquartz wackes was clearly evident in the change from high to low resistivity values. At the eastern end of the profile, the increase in resistivity values indicated the commencement of the porphyrites.

The inhomogeneous Bronze Age dump body extended from E30 to the end of the profile at E64, its thickness varying between ~8 and ~18 m, with the thickest area between E46 and E52. Between E34 and E41 the mediaeval or modern mining dump II was delineated, while the modern refuse dump could be observed between E46 and E52.

A prominent horizontal structure with high resistivity values emerged between E22 and E30 at a depth of about 10–15 m (about 718–728 m a.s.l.), an anomaly also related to a high polarization response (Figure S1b) pointing to the presence of residual mineralization. Possibly, underground mining was attempted from the hole at E30, but this cannot be confirmed on the basis of the present data. There was also an increased polarization response in the limestone between E1 and E10. Although limestone is generally rather non‐polarizable, the proximity of the tectonic transition to the metaquartz wackes means that local polarization anomalies may be explained by the presence of secondary mineralization.

### 4.3 Transverse profile Q6

Transverse profile Q6 (Figure S2) ran from west to east about 30 m south of and parallel to Q2. Borehole KB02 was located 12 m south of measuring point E40. From the beginning of the profile at E1 to point E26, the profile ran across the natural slope of the terrain, while from E26 eastwards, it traversed the visible surface of dump III. At 155 m, it crossed the modern state roadway and ended just before the far embankment at E71.

As in Q1 and Q2, the resistivity imaging results (Figure S2a) showed the transition from the limestones (high resistivity) to the metaquartz wackes (low resistivity) at the beginning of the profile. At the eastern end, the increase in resistivity values from E46 onwards indicated the beginning of the porphyritic rock. The Bronze Age dump body extended from E26 to the end of the profile at E71. The thickness of the dump increased continuously from west to east, reaching a constant thickness of ~10 m at E36. For the most part, the dump was characterized by high resistivity and medium phase values, as expected for materials containing low (but measurable) volumetric contents of iron sulphides and metallic minerals (Bücker et al., 2018, 2019; Flores‐Orozco et al., 2021). A low resistivity anomaly between E56 and E61 corresponded to anthropogenic noise, including the modern roadway between the farmhouse at Gasteil No. 7 and the house at Gasteil No. 12. The low resistivity area between electrodes E32 and E33 represented the steep, hardened forest road which the profile crossed.

Like core KB01 from profile Q2, core KB02 recorded mining area A. In the electrical imaging results, mine A was represented by low electrical resistivity around electrode E36, extending from the surface to a depth of about 33 m, and by elevated phase values (Figure S2b). To the east, the profile was bounded by porphyrite. The two further mining areas interpreted in profile Q2 were also resolved in profile Q6, validating the earlier interpretation by independent processing. In profile Q6, upper mine C was revealed by the high resistivity values observed between electrodes E16 and E25, corresponding to the backfill (width about 25 m and max. depth 18 m). In contrast to Q2, the mine at this point appeared to have relatively steep walls and a slightly trough‐shaped bottom. The high resistivity values suggested that the backfill was composed of natural calcareous alpine material apparently removed from further up the slope, where the expected natural slope debris was conspicuously absent in Q6. The deep mining pit B was resolved by high resistivity values between E26 and E36, representing the backfill, reaching a width of about 30 m and a depth of about 30 m. A small area of elevated phase values appeared at a depth of between 20 and 30 m below E11 at the boundary with the limestone and could be residual mineralization. Other areas with elevated phase values were outlined below E32–E36 and E40–E45. These probably represented residual iron ore in the Bronze Age dumps or veins in the landslip material recorded in KB02.

### 4.4 Transverse profile Q3

**FIGURE 6:**
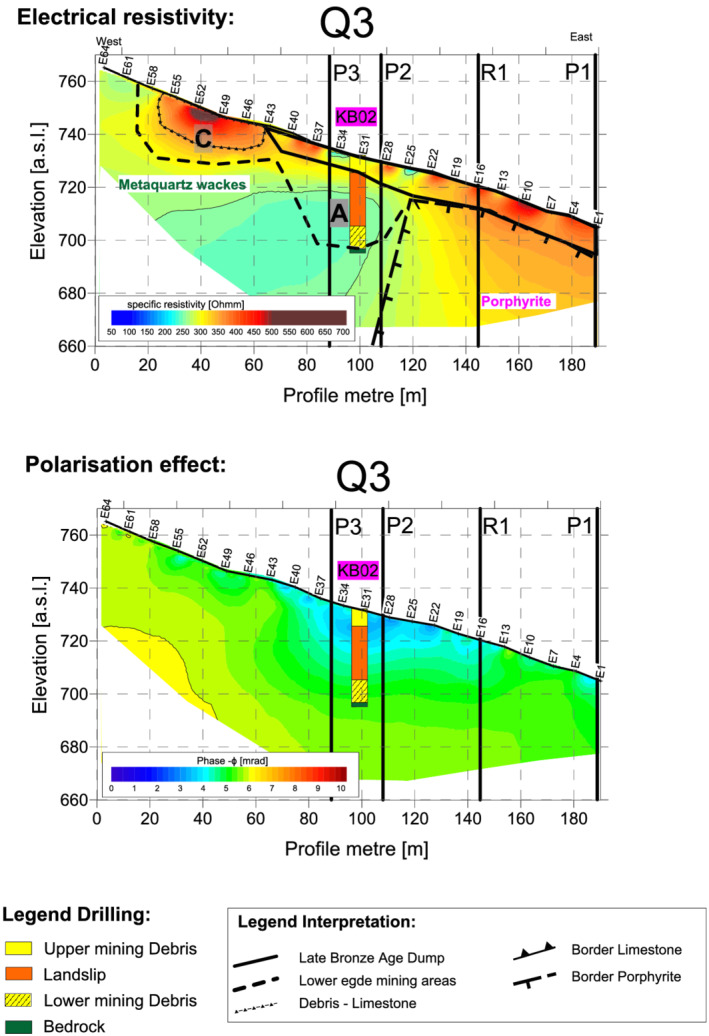
Prigglitz‐Gasteil. Profile Q3: (a) resistivity and (b) induced polarization imaging results [Colour figure can be viewed at wileyonlinelibrary.com]

Transverse profile Q3 (Figure 6) ran from east to west about 10 m south of Q6. Borehole KB02 was located just south of the profile at E32. From E64 at the western end as far as E43, it ran along the natural slope of the terrain. The Bronze Age dump began at electrode E43 and could be traced as far as the eastern end of the profile at E1, right above the cutting for the state road. The transition from limestone (high resistivity) to metaquartz wackes (low resistivity) was not recorded in Q3, probably because it was located farther uphill and to the west (Figure 6a). The increase in resistivity at electrode E25 was interpreted as the boundary between the metaquartz wackes and the porphyrites. Between E56 and E43, a wide trough‐shaped pit was outlined in the metaquartz wackes (width ~40, max. depth 13 m), which was filled with material whose high resistivity values suggested it was local calcareous slope debris. This pit corresponded to the uppermost mining area C in transverse profile Q6.

The Bronze Age dump body extended from E1 to E43 with a consistent thickness of about 8–9 m. For the most part, the dump was characterized by high resistivity and low phase values, probably indicating a porous material with poor iron content, such as calcareous rock (Figure 6b). The near‐surface low‐resistivity area at E34 corresponded to the cutting of the forest road, which intersected the dump here. A second near‐surface anomaly characterized by low resistivity values was visible at E25. This did not have any connection with variations in the terrain and may have been a backfilled excavation cut by Franz Hampl in 1958 (Hampl & Mayrhofer, 1963). Unlike in Q6, the modern roadway, which crossed Q3 at E16, did not show up in the electrical imaging results.

**FIGURE 7:**
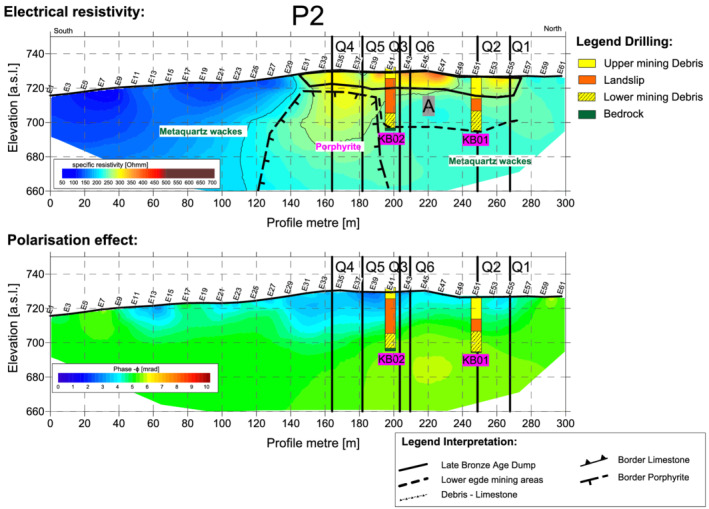
Prigglitz‐Gasteil. Profile P2: (a) resistivity and (b) induced polarization imaging results [Colour figure can be viewed at wileyonlinelibrary.com]

The 35.4 m‐deep mining area A, documented in KB02, was indistinguishable from the metaquartz wackes in the resistivity image (Figure 6). The reason for this was probably the 20.15 m‐thick layer of landslip material, composed of metaquartz wackes, which did not offer enough contrast with the underlying rock to be resolved (Trebsche & Weixelberger, 2022). This explanation also applies to the absence of variations in the polarization images (Figure 7b), which also suggests an absence of metallic minerals.

### 4.5 Transverse profile Q5

Transverse profile Q5 (Figure S3) ran from west to east about 20 m south of and parallel to Q3. Borehole KB02 was located to the north, at a distance of 15 m from the measuring point E28. As in profile Q3, the transition from the limestones (high resistivity) to the metaquartz wackes (low resistivity) was not recorded (Figure S3a). The increase in resistivity values at E31 was interpreted as the boundary with the porphyrites. As in profiles Q3 and Q6, an anomaly associated with high electrical resistivity values was observed between electrodes E4 and E13 and interpreted as corresponding to the backfill material of mining area C, which here appeared as a trough‐shaped pit (width about 25 m, max. depth 19 m).

From E14 onwards, Q5 traversed the Bronze Age dump body, which extended to the end of the profile at E64, above the embankment of the state road. The thickness of the dump was between 5 and 10 m. The dump was characterized by high resistivity and phase values, suggesting a relatively high content of dispersed sulphide minerals, while polarization at 1 hz suggested that the mineral inclusions were relatively coarse (e.g., Bücker et al., 2018; Pelton et al., 1978). The shallow low‐resistivity areas at E37 and E52 may have corresponded to backfilled sections of the excavations conducted by Franz Hampl in 1958 (Hampl & Mayrhofer, 1963). Mining area A, attested by core KB02, could no longer be recognized in the imaging results for profile Q5. As the porphyrites extended far to the west of the profile, Q5 may already have lain south of the extraction boundary. Nonetheless, an anomaly characterized by some of the highest polarization values was observed (Figure S3b) between E19 and E28, at a depth of between 10 and 30 m, pointing to residual mineralization (copper, iron). Some parts of the dump body itself also showed elevated phase values, tending to confirm the presence of such metals.

### 4.6 Transverse profile Q4

Transverse profile Q4 (Figure S4) ran from east to west and was the southernmost transverse profile. From E61, at the western end, to E29, it ran along the natural slope, cut by the modern logging road (characterized by anomalies with low electrical resistivity values between electrodes E54 and E53). A second shallow anomaly was found between electrodes E39 and E48, which could not be explained by any surface feature. At E29, an anomaly with low electrical resistivity values was caused by the foundations of an elevated water tank supplying the Gasteil No. 7 farmhouse. Below this tank, a dump cone was clearly visible in the terrain. The profile continued downhill across the mining dump and ended at the top of the embankment of the state road. In the resistivity results (Figure S4a), the transition from the limestones (high resistivity) to the metaquartz wackes (low resistivity) was evident at E39. At the eastern end of the profile, the increase in resistivity values from E24 indicated the commencement of the porphyrites.

The Bronze Age dump body extended downslope from E29 to the eastern end of the profile at E1. The thickness of the dump was between 5 and 8 m, thinner than in the northern profiles. The dump was characterized by very high resistivity and low phase values, indicating a negligible content of metallic minerals. A low resistivity anomaly was clearly visible in the dump between electrodes 17 and 20, which was consistent with the backfilled excavation areas 2–6 from the excavations conducted between 2011 and 2013 (Trebsche, 2014a, 2014b).

The trough‐shaped depression from E38 to E29 was interpreted as a continuation of the uppermost mining area C, the high electrical resistivity values being associated with the backfill of slope debris. In this profile, mine C revealed a width of ~38 m, and a maximum depth of nearly 20 m. An area of elevated phase values (Figure S4b) was evident between E31 and E22. This wide anomaly, which also extended into the area of the porphyrites, could not be clearly interpreted.

### 4.7 Longitudinal profile P2

**FIGURE 8:**
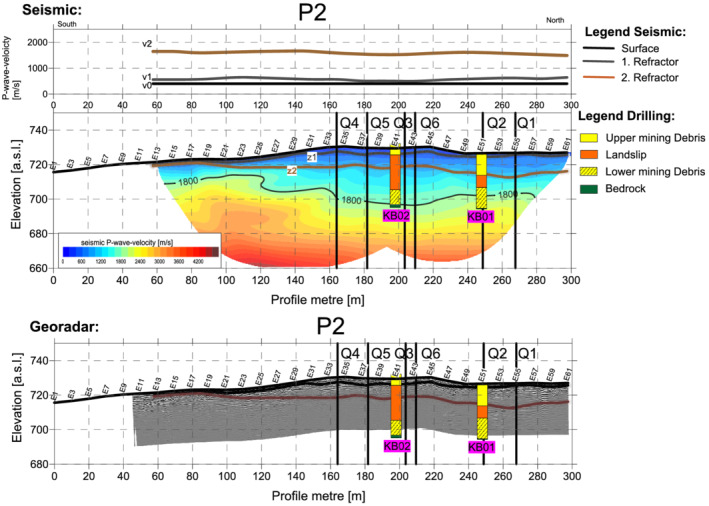
Prigglitz‐Gasteil. Profile P2: (a) refraction seismic and refraction tomography and (b) Georadar [Colour figure can be viewed at wileyonlinelibrary.com]

Profile P2 (Figures 7 and 8) ran from south to north, approximately parallel to the elevation lines of the slope. Borehole KB01 lay exactly on the profile at E41, while borehole KB02 was located 11 m to the west of electrode E51. The resistivity imaging result (Figure 7a) showed the low resistivity area of the metaquartz wackes in the southern half of the profile (between E1 and E30), with slightly higher resistivity values to the north corresponding to the porphyrites (E30 to about E39). Further north still, the model again revealed the lower resistivity values corresponding to the metaquartz wackes. The transition from the metaquartz wackes (low resistivity) to the somewhat higher resistivity values of the porphyrites between E27 and E39 was not, however, as pronounced as in longitudinal profile P3.

The inhomogeneous Bronze Age dump body extended from electrodes E30 to E57, with a thickness of between 8 and 11 m. The resistivity values in this area were much higher than those visualized in other profiles for the dump. Consistent with these observations, the polarization image (Figure 8b) revealed phase values in the lower range for the dumps. These observations may point to an efficient extraction of the metals in this area, with the dumps being associated with the host rock.

Mining area A, documented by core drillings KB01 and KB02, was indistinguishable from the metaquartz wackes in the electrical images. However, it could be clearly seen in longitudinal profile P2 that the porphyrites probably formed the southern boundary of the mine.

The seismic analysis (Figure 8a) revealed a three‐layer model, as in profile Q2. The first layer could be assigned to the topsoil. At a depth of 1–4 m, the second layer could be assigned to the slope debris or the stockpile material (loose rock). At the beginning of the profile, to the south (E13–E25), this layer lay at a depth of ~2–6 m. Thereafter, it dropped to a depth of about 10 m, where it remained almost constant to the end of the profile in the north. As far as E30, the refraction interface represented the transition from the slope debris/weathering layer to the bedrock of the metaquartz wackes. From E30 onwards, it could be interpreted as the transition from the lower edge of the dump material (hanging dump) to the original ground surface.

Below the second layer, the velocity distribution showed an increase in velocity from the 1800 m/s isoline, which was interpreted as the transition to the bedrock (metaquartzites and porphyrites).

The data from georadar (Figure 8b) showed a penetration depth of only about 5–8 m and did not provide any information about the mining areas or the dump thickness. These results will not, therefore, be further discussed.

### 4.8 Longitudinal profile P3

The westernmost longitudinal profile P3 (Figure S5) ran from south to north at a distance of about 15 m from longitudinal profile P2. Borehole KB02 was located about 7 m to the east of E30. In the resistivity model (Figure S5a), the transition from low resistivity to slightly higher resistivity values between E51 and E36 evidently corresponded to the transition from the metaquartz wackes to the porphyrites. The Bronze Age dump body extended from E50 to the beginning of the profile at E1. The thickness of the dump ranged from 8 to 15 m, increasing towards the north. At the intersection with transverse profile Q1 (E11 to E6), the mediaeval/modern‐period dump II was still visible. Resistivity values here were higher than those in previous profiles, while the polarization response revealed comparatively lower phase values.

The mining area A between E36 and E16 was characterized here, as in profile Q2, by low resistivity and elevated phase values, probably indicating the presence of sulphides. The southern boundary of this mining area was resolved at the steep edge of the porphyrite. An anomaly was resolved between electrodes E21 to E16, characterized by high resistivity and low phase values, extending to a depth of more than 20 m. The interpretation of this anomaly was challenging; it could have been related either to the northern wall of the mine or a remaining part of the bedrock. Further ground‐truth data is required for a proper interpretation, especially as this anomaly was not resolved in the intersecting profile Q2. In the 1950s, Hampl and Mayrhofer recognized a pit mouth at this location, (Hampl & Mayrhofer, 1963, p. 59), which was not visible during our measurements because of the vegetation. Between electrodes E56 and E43, at a depth of approximately 10 m, there was an area of elevated phase values (Figure S3b) at the transition from the metaquartzites to the porphyrites, which we interpreted as a secondary mineralization, similar to the one observed in Q1. This mineralization was related to the anomaly, also associated with a high polarization response, resolved in profile Q4 (E31 to E21).

### 4.9 Longitudinal profile R1

Profile R1 (Figure S6) ran from south to north, parallel to P2, directly along the driveway to the timber yard and the house Gasteil No. 12. Around electrode E21, as in the previous longitudinal profiles, imaging results (Figure S6a) showed the transition from relatively high to low resistivity values, corresponding to the transition from the porphyrites to the metaquartz wackes. The Bronze Age dump body extended from E66 to E5. The thickness of the dump increased continuously above the porphyrites from south to north, from 6 m to a maximum of 19 m (at E16). Resistivity and phase values varied widely within the dump, indicating a variable quantity of iron sulphides in the dumps. Mining area A was no longer evident here. The data from the georadar (Figure S6b) showed a penetration depth of ~5–8 m, but no information about the mining areas or the dump thickness and will therefore not be further discussed.

### 4.10 Longitudinal profile P1

The easternmost longitudinal profile P1 (Figure S7) ran from south to north along the western edge of the state road. The resistivity imaging results (Figure S7a) showed the transition from the low resistivity associated with the Radschiefer shales to the higher resistivity values of the metaquartz wackes at E52. According to cross section Q2, some high resistivity values due to the porphyrites should still be visible here; however, no clear transitions in the resistivity model (to higher values) can be observed. This may be explained by the fact that profile P1 is located exactly at the boundary between the porphyrites and the Radschiefer shales or metaquartz wackes. The change in the geology on either side of the profile limited the application of the method, which assumes no changes in the third dimension; that is, the materials are consistently parallel to the imaging plane. When the current was injected into the ground, it flowed preferentially through the areas with lower resistivity values. Accordingly, no resistivity data was collected at P1 from the porphyrites, which were therefore not mapped in the imaging results.

The Bronze Age dump body could be divided here into three clearly separated areas. The southernmost area was located between E19 and E37 and had a thickness of 13 m. The middle area was located between E39 and E55 and reached a thickness of 17 m. The northernmost area extended from E57 to E63 with a maximum thickness of 8 m. The resolved high resistivity values and the low polarization response suggested that the tailings were associated with a minimal concentration of sulphides (Figure S7b).

## 5 DISCUSSION

### 5.1 Geological units

**FIGURE 9:**
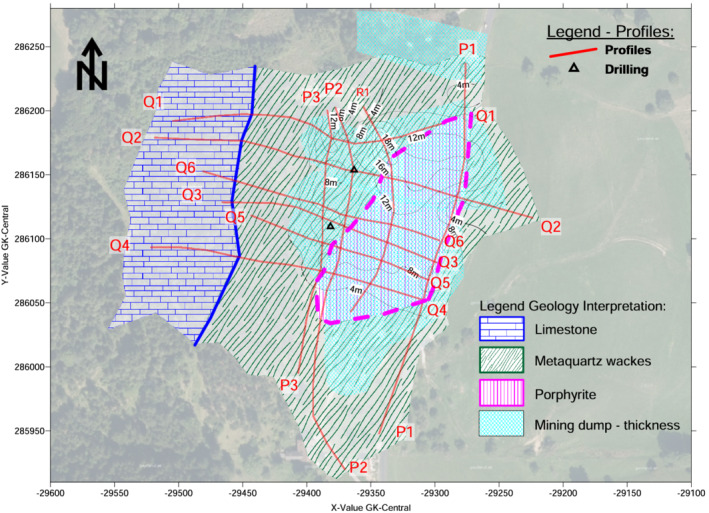
Prigglitz‐Gasteil: Interpretation of geological units and interpolated thickness of the mining dumps [Colour figure can be viewed at wileyonlinelibrary.com]

Figure 9 shows the areal distribution of the geologic units, excluding the overlying anthropogenic structures of the mining areas and dump bodies. At the western side of the study area, calcareous alpine rocks were present, clearly separated from the metaquartz wackes, which belonged to the graywacke zone. This boundary resulted from the tectonic movement or overthrust of the limestones over the graywacke zone (Herrmann et al., 1992). The geological contact ran in an approximately north–south direction. Within the metaquartz wackes, a block of porphyrites was intercalated.

### 5.2 Mining dumps

Figure 9 also depicts the areal extent of the mining waste heaps and the underlying mined areas. The isolines depicted within the dumps indicate their thickness.

The thickest dumps were located in the area of the modern timber yard at Q1, where they reached a thickness of more than 18 m. The sand pit and the log yard were apparently constructed on top of the largest dump. In total, the largest dump extended over an area of approximately 160 m × 180 m. It could be resolved into two separate dump cones at the lower eastern end (in profile P1).

A smaller dump (min. 40 m × 100 m) was resolved at the northern edge of the study area in the electrical imaging results of the longitudinal profile P1.

### 5.3 Late Bronze Age mine workings

**FIGURE 10:**
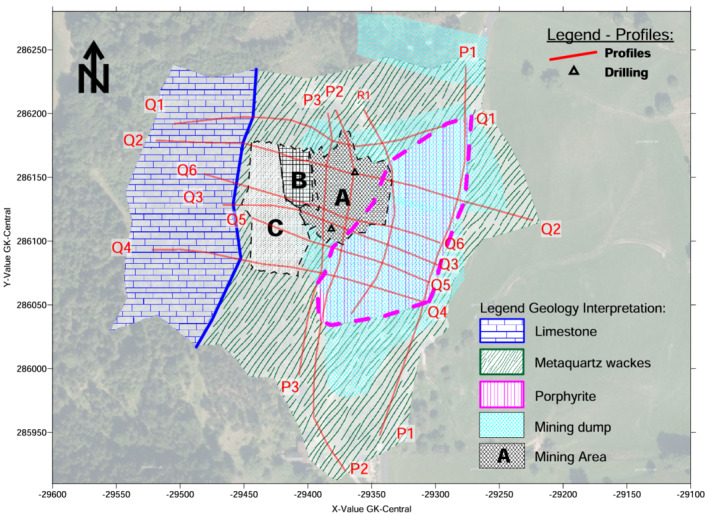
Prigglitz‐Gasteil: Interpretation of mining areas A–C [Colour figure can be viewed at wileyonlinelibrary.com]

**FIGURE 11:**
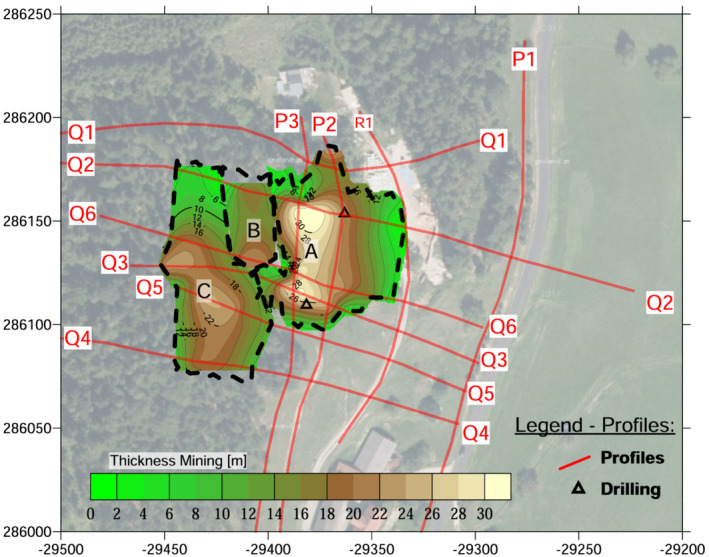
Prigglitz‐Gasteil: Interpolated depth of the mining areas A–C [Colour figure can be viewed at wileyonlinelibrary.com]

**FIGURE 12:**
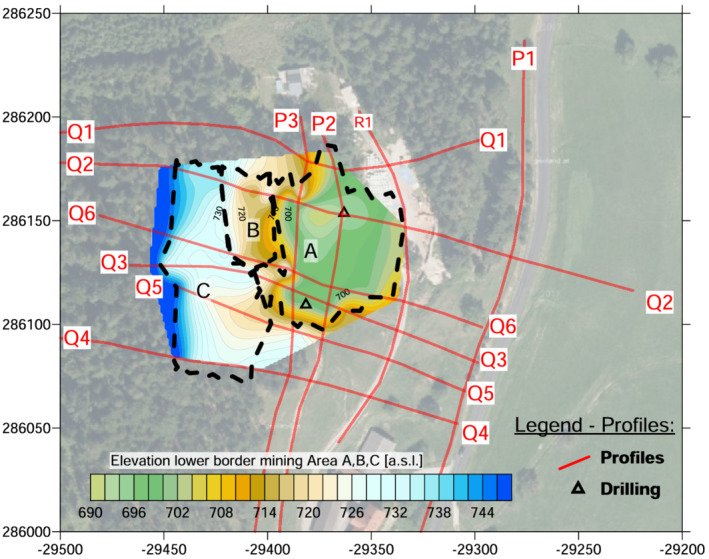
Prigglitz‐Gasteil: Interpolated lower edge of the mining areas A–C [Colour figure can be viewed at wileyonlinelibrary.com]

The mine workings, completely covered by the dumps or slope debris, were divided into three areas (Figures 10, 11, 12): A–C. The lowest mining area, A, was resolved in imaging results for profiles Q2, Q6, Q3, and Q5; it did not show up in the northernmost profile, Q1, or the southernmost profile, Q4. The north–south extension could be readily seen in longitudinal profiles P3, P2, and R1. From the imaging results, it could be ascertained that mine A extended over a length (N‐S) of 60–65 m and a width (E‐W) of 30–40 m, and reached a maximum depth of 33 m below the present surface. It was a large kidney‐shaped pit in plan with steep walls to the north, south and west. The bottom was approximately horizontal, with a deeper depression in the northern half of the pit. The interpretation of the electrical anomalies was validated by the stratigraphy recovered in the drilling core KB01 (Trebsche & Weixelberger, 2022). To the east and south, the mining area was bounded by the porphyrites, which formed a steep wall. The western boundary was only indistinctly visible in the electrical images. This could probably be explained by the mining subsidence event around 920 BC that resulted in a large‐scale landslide (Trebsche & Weixelberger, 2022). The landslip material, up to 20 m thick, buried a considerable part of the already abandoned and partly backfilled mining area.

Mining area B was retrieved in the electrical images of profiles Q2 [E37–E46] and Q6 [E26–E36] but was not visible in profile Q1, which was located further north. There may have been some indications of it visible at the southern end of profile Q3 [E40–E37], but it was certainly no longer present in profile Q5. No longitudinal profiles were measured in this area to confirm our interpretation. The width (N‐S) varied from around 45 m to not more than 65 m. From east to west, the mine measured a maximum of 30 m in profile Q6, but only about 7 m at the southern end of profile Q3. The ground plan of the north–south oriented, that is, slope‐parallel pit was elongated, the bottom trough‐shaped, its walls steep. As far as can be seen in cross sections Q2 and Q6, it reached a depth of ~23–30 m.

The uppermost mining area, C, was resolved in the imaging results of profiles Q2 [E27–E37], Q6 [E16–E25], Q3 [E56–E43], Q5 [E4–E13] and Q4 [E38–E29]; longitudinal profiles were not measured in this forested area of the site. It was not mapped in the northernmost profile, Q1. To the south, it may have continued beyond Q4, the southernmost of our geophysical survey lines. Thus, its north–south extent could be approximated to at least 90 m, while its width (measured east–west) varied between 25 and 40 m. Its depth varied between about 12 and 20 m. The mine ran parallel to the slope immediately below the eastern edge of the alpine limestone. The Bronze Age dump was visible adjacent to the eastern edge of the mining area in all cross sections. Imaging results for profile Q2 suggested that mining area C was buried under slope debris consisting of calcareous alpine material (corresponding to the high resistivity values observed), probably by erosion, which is why it is no longer evident in the terrain today.

In summary, the geophysical measurements provided evidence for three open‐pit mine workings for copper ore on the slope of the Gahns mountain. They were laid out in the Late Bronze Age; more precisely, in the 11th to 9th centuries BC, as shown by radiocarbon dating of organic find material from core drillings KB01 and KB02 and by archaeological excavations conducted in 1956, 1958 and 2010 to 2014 (Trebsche, 2015; Trebsche & Weixelberger, 2022).

Opencast mining was a common technique for exploiting mineral resources during the Bronze Age (e.g., the copper mines of Great Orme in Wales; O'Brien, 2015; Timberlake, 2017). Paradoxically, in the Alps, prehistoric underground mines have been much better investigated, because most prehistoric mines were discovered in the 19th century during modern underground mining activities. Opencast mines, on the other hand, were often backfilled and are thus more difficult to detect. The use of geophysical methods, as in the present study, is therefore critical for maximizing the outcome of any investigations. It is generally assumed that underground ore mining originated, in most cases, from an open‐pit mine (e.g., O'Brien, 2015, pp. 169, 200–203). In fact, in the Mitterberg region in Salzburg, geophysical prospections have proved that the Brandergang underground mine began as an open pit (Pingenbau; Stöllner et al., 2006, pp. 123–129; Thomas, 2018, pp. 398–399).

Based on mining considerations, as well as on the stratigraphy of the backfill layers, the three mining areas could not have existed at the same time, and probably succeeded each other. The most plausible sequence is (1) mine A, (2) mine B and (3) mine C. The ore deposits were progressively mined from lower to higher levels on the mountain slope, so that in each case the older mining area was filled up with the dump material from the younger mine. According to the evidence from the cores, there was also a mining subsidence event in which a landslide buried a portion of the already abandoned mine A. The uppermost, youngest mining area, C, is thought to have been gradually filled with natural slope debris after it was abandoned, making it no longer visible on the surface today.

Regarding the volumes of the three above‐mentioned mining areas (see Table 3), the latest, C, was the largest, at approximately 88,000 m^3^, followed by the earliest, A, at ~76 000 m^3^, with the comparatively small mining area B in between (~14 000 m^3^). The total of the mined volumes at Prigglitz‐Gasteil (178 000 m^3^) corresponds well with the calculated total volume of the mining dumps (216 000 m^3^). The volume increase of ~21% can be explained by the swell factor from the bank volume of the solid rock to the loose volume of the mining dumps. Furthermore, the volume recorded for the mining dumps did not include the volume of the landslip layer, which could not be delineated in the geophysical prospections.

**TABLE 3:** Prigglitz‐Gasteil: Volume calculation of mine workings and dumps

Volume	m^3^
Dump	216 220
Mine working A	75 927
Mine working B	14 153
Mine working C	88 199
Mine workings total	178 279

Our new volume calculations, based on the three‐dimensional geophysical prospection, exceed by far the older figures, based on superficial estimates, by Hannes Mohr (60 000 to 80 000 m^3^; Mohr, 1958). The rough estimates (75 000 to 110 000 m^3^) by Günther Weixelberger, made after the first pile core probing campaigns at the site in 2014, were also too low, because the underlying bedrock was not reached across all of the study area (Trebsche & Weixelberger, 2022). These results from Prigglitz‐Gasteil can be compared with the roughly contemporaneous Middle to Late Bronze Age salt mine at Hallstatt (Upper Austria). There, similar volumes are reported to have been mined, even underground, with the dimensions of one mining gallery being ~100 m × 40 m × 18 m = 72 000 m^3^ (Kowarik et al., 2015).

### 5.4 Ore mineralization

From the IP imaging results, it could be deduced that the mineralization was mainly located within the metaquartz wackes, some of it right at the boundary with the limestone, but not in the calcareous rocks themselves. The eastern boundary of the mineralization was formed by the porphyrites. Residual mineralization was inferred in two places, according to the polarization images. The first, larger zone, which could not have escaped the attention of the Bronze Age miners, was found directly south of mining area A, at a depth of 10–30 m (Figure S3b; Q5 [E19–E28]). However, the miners did not extend mine A to the south, preferring to move to a higher level in the terrain (mine B). The reason was probably that the mineralization was no longer accessible due to the above‐mentioned subsidence, which backfilled the southern part of pit A with landslip material up to 20 m thick. This mineralization south of pit A is also outlined in Q4 (Figure S4b; [E31–E19], ~700–720 m a.s.l.) and P3 (Figure S5b; [E56–E43], c. 708–720 m a.s.l.). Mining area C reached this zone from the west but was unable to follow it further east (downslope), probably because too much dump material had already been deposited there. The second mineralization zone lay at the very western edge of the metaquartz wackes, at the boundary with the thrust calcareous‐alpine rocks. Ore remnants were evident in Q2 (Figure 4b; [E19–E25], 740–750 m a.s.l.) and Q6 (Figure S2b; [E7–E12], 730–745 m a.s.l.) but were left standing in mining area C. The reason for this was probably that the slope of the calcareous alpine rock above the edge of the mine was already too steep and no further mining failure could be risked.

The Bronze Age dump and the backfill, especially of mining area A, were characterized by their inhomogeneous composition, which in some places indicated intercalated ores, as resolved in the polarization images. The high ore content of the dumps can be simply explained by the fact that during the Bronze Age only the copper ore was selected, while the iron ore (siderite, limonite) was dumped because it could not be beneficiated using Late Bronze Age smelting technology (Turck et al., 2019, with further references).

### 5.5 Mediaeval/modern period shafts?

The geophysical surveys presented in this study also provided evidence for the function of two possible mouth holes that Franz Hampl was able to visually map in the 1950s (Hampl & Mayrhofer, 1963, pp. 59, 71, fig. 11). At the time of the measurements (2017 and 2018), the mouth holes were no longer visible at the site due to dense vegetation cover and the accumulation of debris at the surface. Nonetheless, they were resolved in the geophysical images as shallow anomalies. The two mouth holes probably belonged to a small mediaeval or modern‐period test pit for iron ore. The northern mouth hole is actually located over an ore body, as indicated by elevated polarization values in Q1. The associated dump III, which is easily recognizable, superficially, as a young dump above the Bronze Age dumps, was clearly imaged in Q1 and P2. Its extent and volume were very small compared to the thick Bronze Age dumps.

The 5–8 m deep mouth hole observed by Hampl (Hampl & Mayrhofer, 1963, pp. 59, 71, fig. 11 ) at the western end of terrace T7 (‘Planum B’ according to Hampl; cf. Figure 1) coincided in location with a vertical structure in longitudinal profile P3 at E15. This could be the mouth of a shaft that led into mineralized areas within the metaquartz wackes. No associated dump was visible superficially.

## 6 CONCLUSIONS

With the help of electrical imaging techniques, namely, electrical resistivity and induced polarization tomography, combined with seismic refraction tomography, it was possible to reconstruct an open‐pit copper‐ore mine of the Late Bronze Age. No evidence of open‐pit mining was superficially discernible in the morphology of the terrain, except for the spoil heaps. As far as the terrain and the forestation of the site allowed, a regular orthogonal grid consisting of a total of six longitudinal and four transverse profiles was laid out over an area of about 330 × 300 m. The rectangular gridding of the profiles allowed a three‐dimensional interpretation of the geological units, the mining dumps, the mining areas and the residual mineralization.

Decisive for the interpretation of the geophysical measurements was the availability of materials recovered from two deep cores. The use of geophysical measurements and deep drilling is rare in archaeological studies, making our study a possible standard for future investigations. In particular, the deep cores provided critical information about the stratification, which aided in the interpretation of the geophysical anomalies. Without the control or verification provided by the cores, mining area A could not have been correctly identified. Additionally, the archaeological finds recovered from the cores provided the opportunity for radiocarbon dating, allowing the open pits to be accurately dated to the Late Bronze Age (11th–9th century BC).

The applied geophysical methods provided different information for the reconstruction of the site: the electrical resistivity images provided the best clues for locating the geological units and the dumps, the seismic tomograms resolved the transition from the dump or backfill layers to the underlying bedrock, and the polarization imaging results revealed residual mineralization. The georadar measurements, on the other hand, did not contribute to the interpretation of the prehistoric mining features owing to the limited depth of penetration.

Based on our investigations with direct and indirect methods, it was possible to develop a hypothetical model of open‐pit mining for copper ore that developed in three phases. Pit A, probably the oldest pit, ran parallel to the slope for a length of 60–65 m, with a width of 30–40 m and a maximum depth of 33 m below the present surface. After its abandonment, mining continued one level higher in mine B (length 45–65 m, width 7–30 m and depth 23–30 m). The overburden from area B, as well as landslip material from a subsidence event, filled the lower mining area A during this phase, as was evident from the cores. In the third phase, mining moved even further upslope and continued in area C (length min. 90 m, width 25–40 m, depth 12–20 m). When all accessible mineralization had been extracted, apart from two small remnants, mining area C was abandoned and finally filled in naturally by erosion.

To conclude, the aforementioned combination of geophysical prospecting methods and core drilling proved successful for archaeological purpose. It can be applied for reconstructing simple and complex remnants of prehistoric mining, in the form of pits, open pits, or underground workings, especially where access to prehistoric features via modern mines is no longer possible. To improve future prospections, a denser and more regular layout of the survey grid (with equidistant survey profiles) and the use of seismic blasts with a greater penetration depth would be useful. In the Bronze Age mining area of Prigglitz‐Gasteil, further core drilling may help to verify the proposed archaeological model, especially in mining areas B and C, as well as an extension of the measuring grid to the north and south to fully cover the periphery of the mining area and to make the volume calculations more precise. In future, research combining geophysical prospection and core drilling and laboratory measurements of the sediments recovered should be used to gain more information about the chemical composition of the materials (e.g., their iron and copper content) as well as their associated electrical properties (electrical resistivity and polarization).

## CONFLICT OF INTEREST

The authors declare that they have no known competing financial interest or personal relationships that could have influenced the work reported in this article.

## Supporting information


**Figure S1.** Prigglitz‐Gasteil. Profile Q1: A resistivity, B induced polarization imaging results

**Figure S2.** Prigglitz‐Gasteil. Profile Q6: A resistivity, B induced polarization imaging results

**Figure S3.** Prigglitz‐Gasteil. Profile Q5: A resistivity, B induced polarization imaging results

**Figure S4.** Prigglitz‐Gasteil. Profile Q4: A resistivity, B induced polarization imaging results

**Figure S5.** Prigglitz‐Gasteil. Profile P3: A resistivity, B induced polarization

**Figure S6.** Prigglitz‐Gasteil. Profile R1: A resistivity and induced polarization imaging results, B Georadar section

**Figure S7.** Prigglitz‐Gasteil. Profile P1: A resistivity, B induced polarization imaging results

## Data Availability

The data that support the findings of this study are available from the corresponding author upon reasonable request.
