# Supplementary figures and images for: Combining geophysical prospection and core drilling: Reconstruction of a Late Bronze Age copper mine at Prigglitz‐Gasteil in the Eastern Alps (Austria)
Source: Archaeol Prospect. 2022 Aug 2;29(4):557–77. doi: 10.1002/arp.1872 (PMC10087026; doi:10.1002/arp.1872)

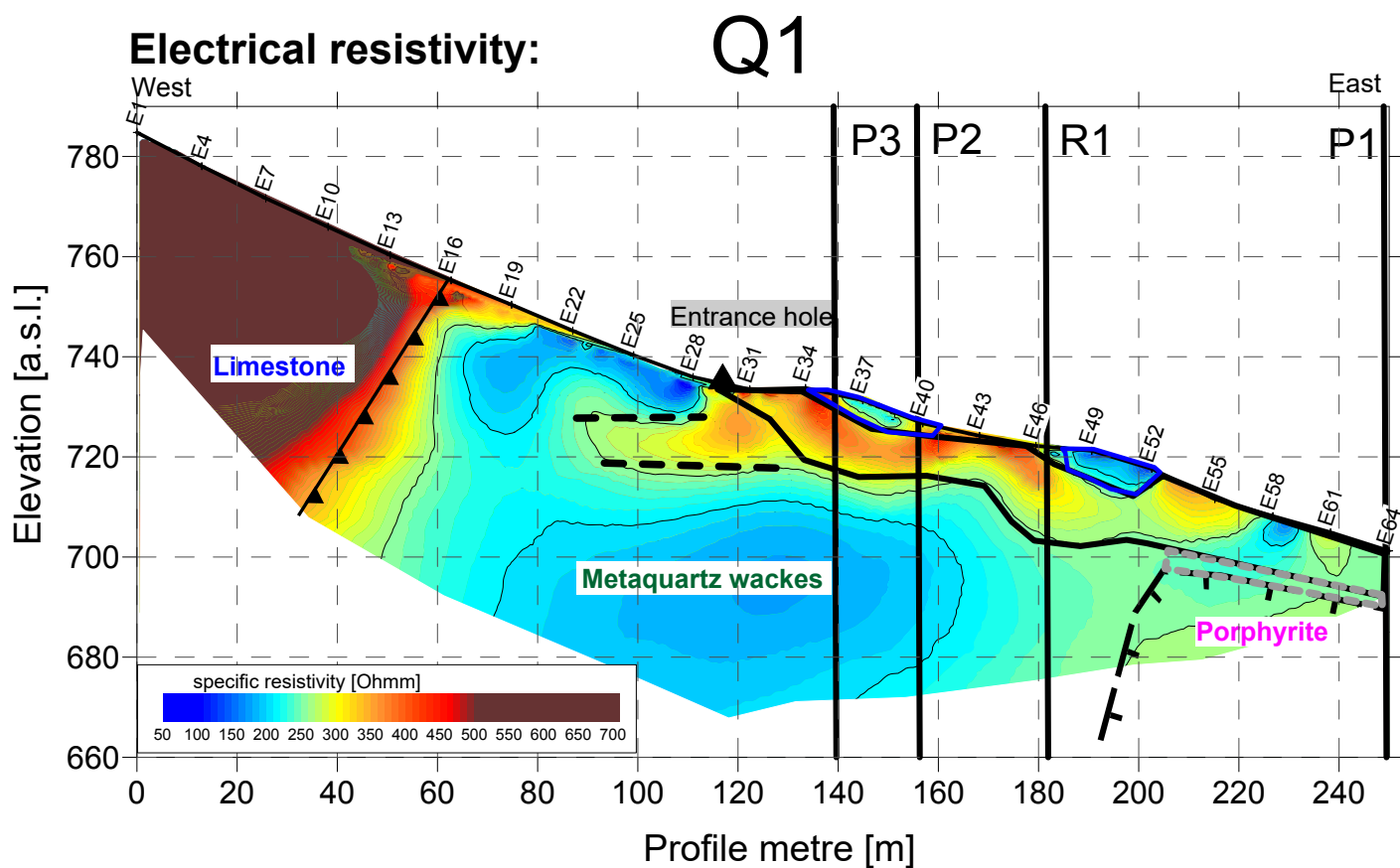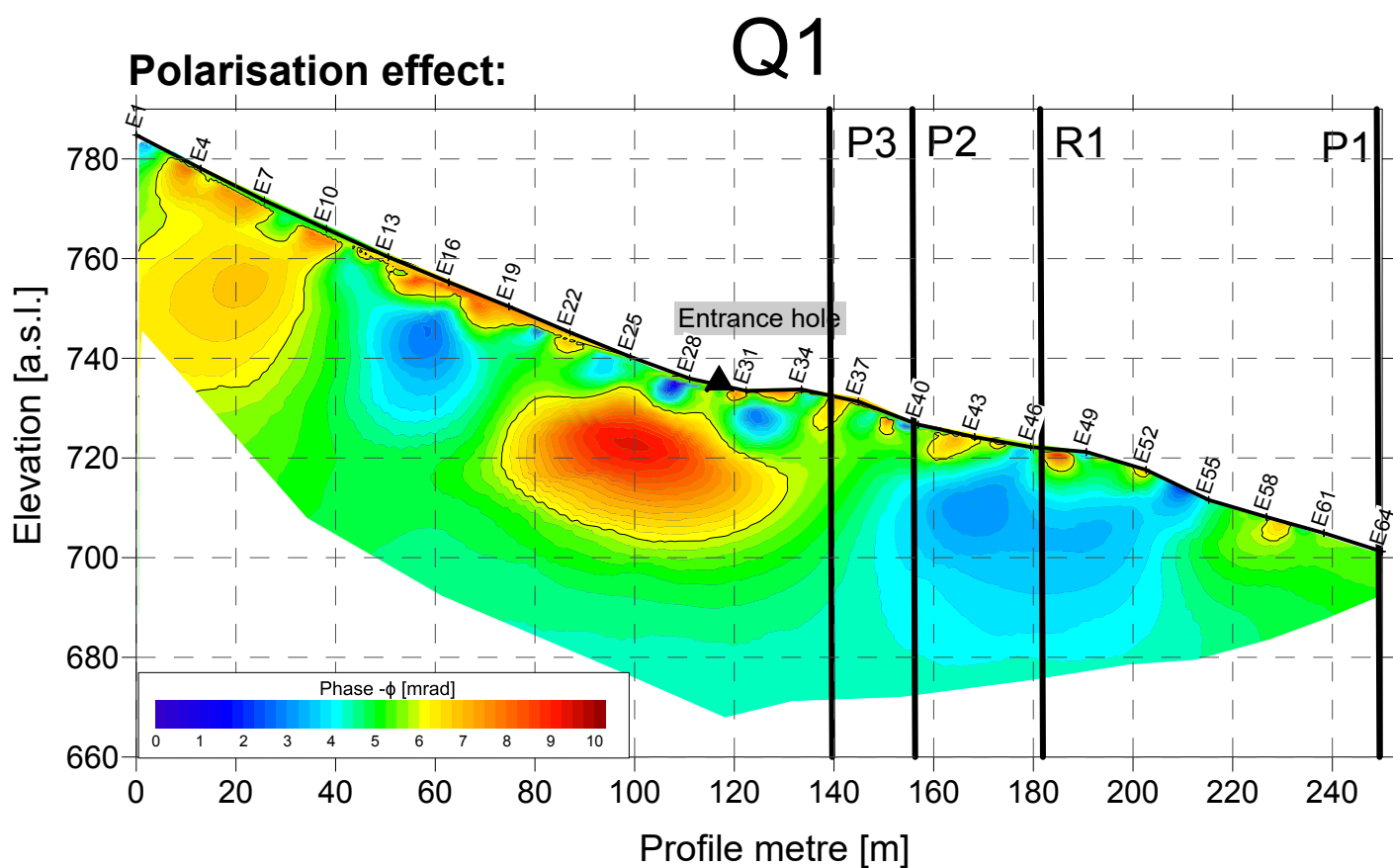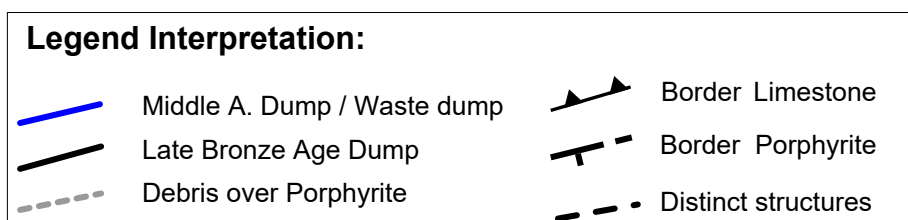

Supplement: Supplementary file 1 — Figure S1. Prigglitz‐Gasteil. Profile Q1: A resistivity, B induced polarization imaging results [file ARP-29-557-s003.pdf]

Electrical resistivity:

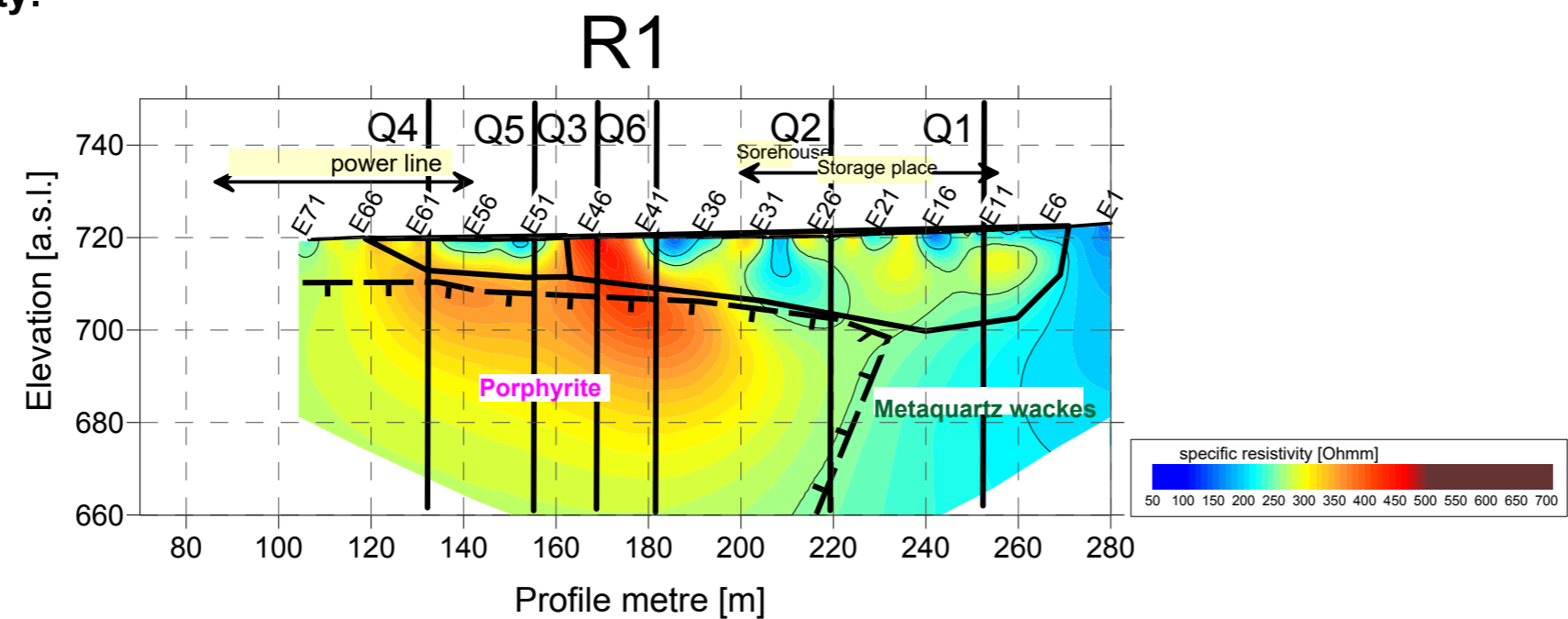

Polarisation effect:

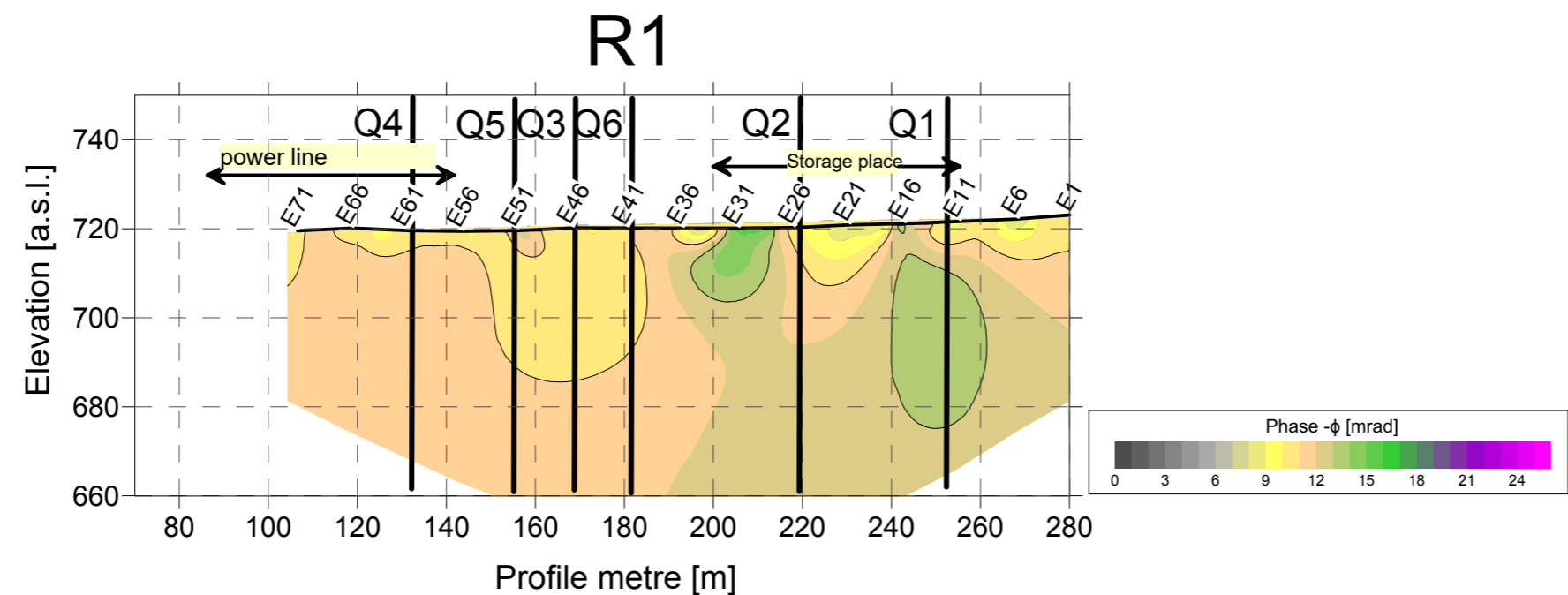

Georadar:

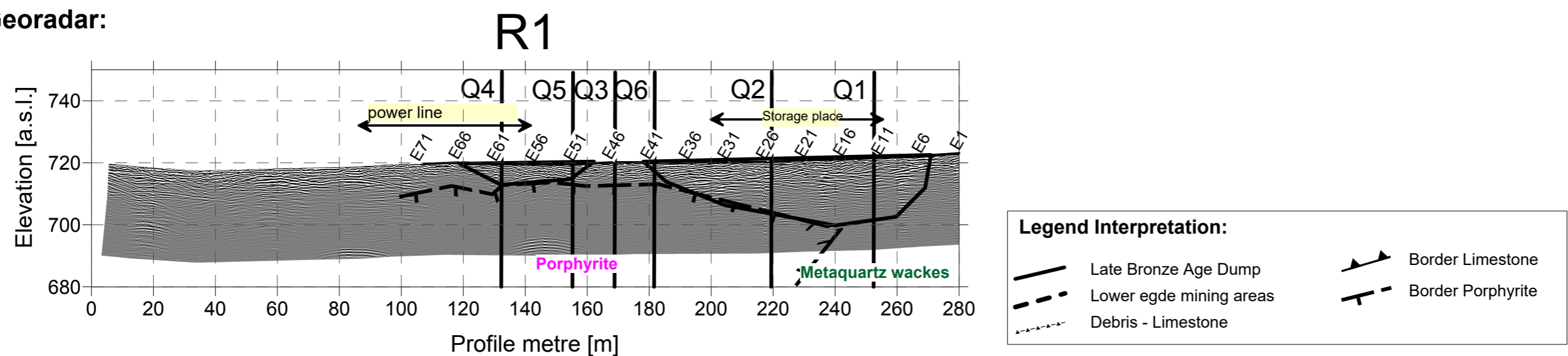

Supplement: Supplementary file 6 — Figure S6. Prigglitz‐Gasteil. Profile R1: A resistivity and induced polarization imaging results, B Georadar section [file ARP-29-557-s005.pdf]
