# Supplementary material for: Combining geophysical prospection and core drilling: Reconstruction of a Late Bronze Age copper mine at Prigglitz‐Gasteil in the Eastern Alps (Austria)
Source: Archaeol Prospect. 2022 Aug 2;29(4):557–77. doi: 10.1002/arp.1872 (PMC10087026; doi:10.1002/arp.1872)

Electrical resistivity: Q5

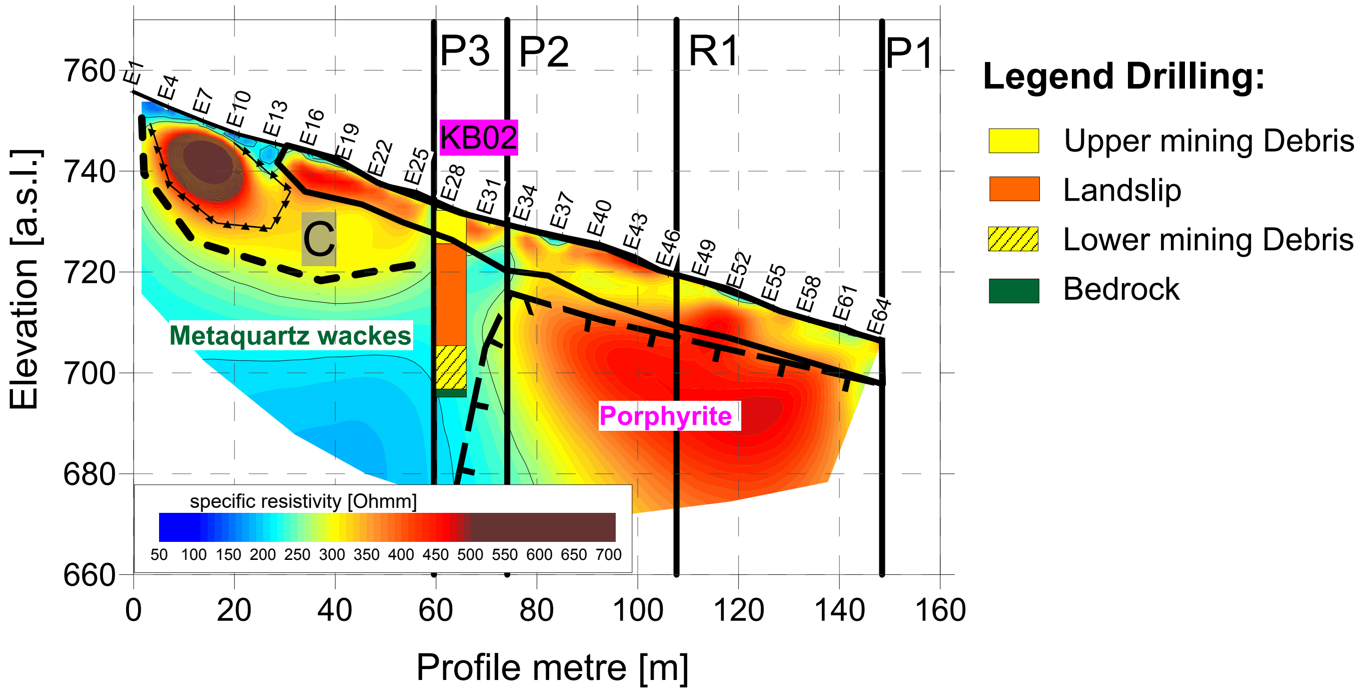

Polarisation effect: Q5

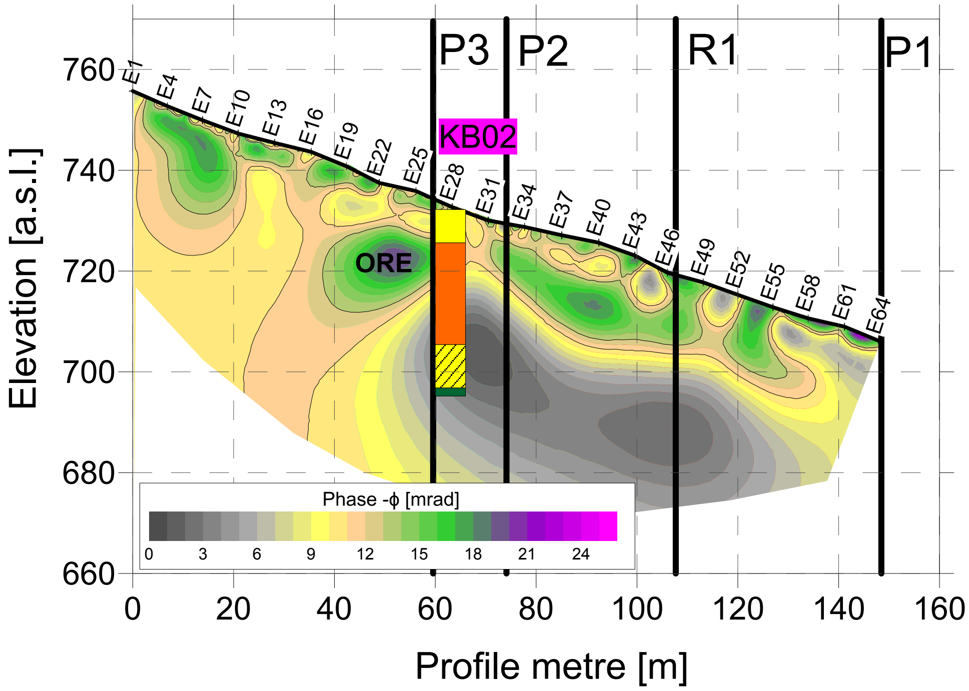

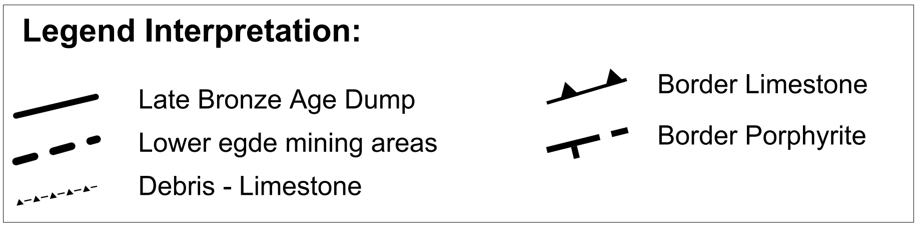

Supplement: Supplementary file 3 — Figure S3. Prigglitz‐Gasteil. Profile Q5: A resistivity, B induced polarization imaging results [file ARP-29-557-s006.pdf]
