# Supplementary material for: Combining geophysical prospection and core drilling: Reconstruction of a Late Bronze Age copper mine at Prigglitz‐Gasteil in the Eastern Alps (Austria)
Source: Archaeol Prospect. 2022 Aug 2;29(4):557–77. doi: 10.1002/arp.1872 (PMC10087026; doi:10.1002/arp.1872)
Supplement: Supplementary file 4 — Figure S4. Prigglitz‐Gasteil. Profile Q4: A resistivity, B induced polarization imaging results [file ARP-29-557-s007.pdf]

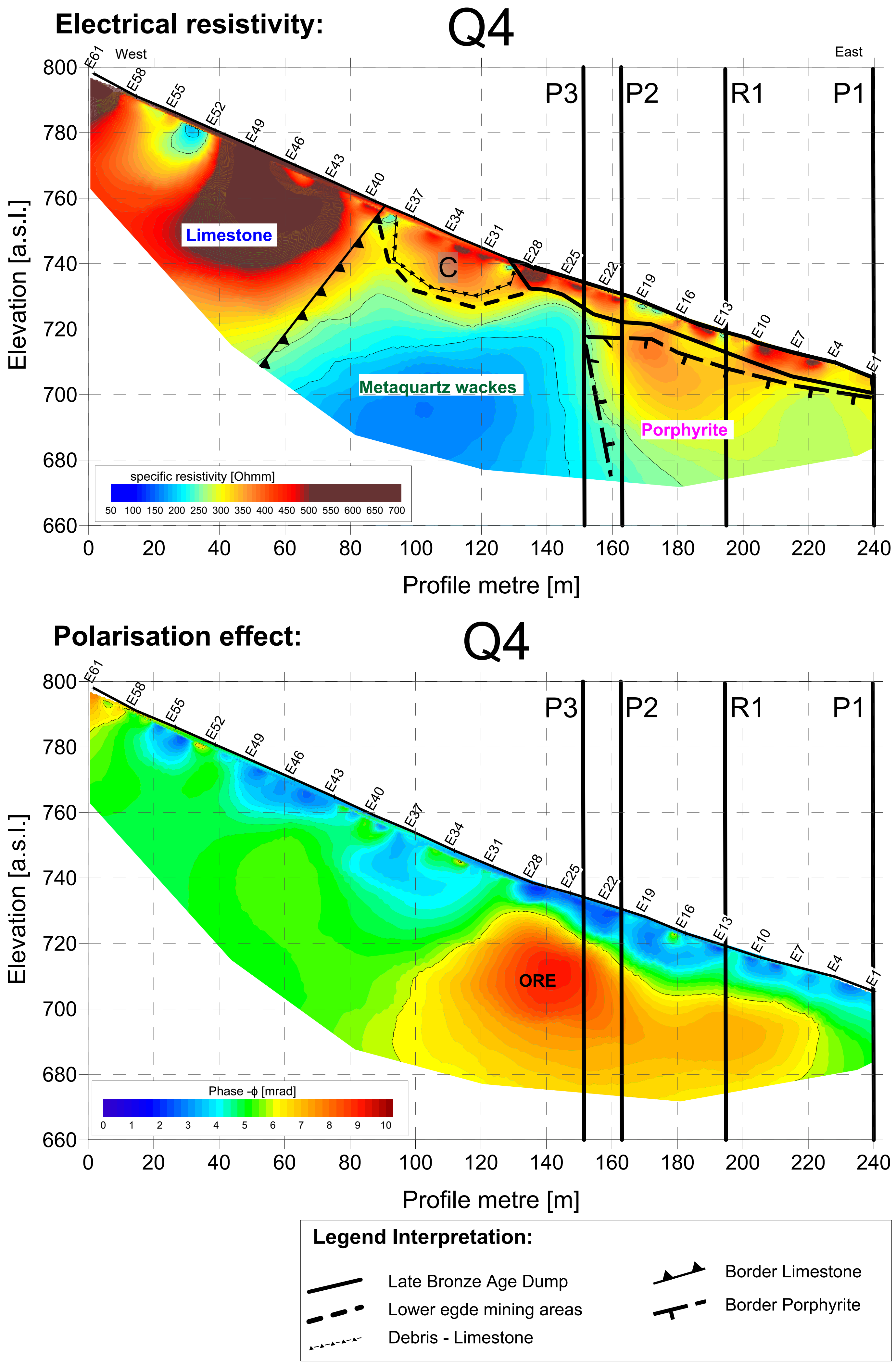

Electrical resistivity:
Q4
West
East
P3
P2
R1
P1
Limestone
C
Metaquartz wackes
Porphyrite
specific resistivity [Ohmm]
50 100 150 200 250 300 350 400 450 500 550 600 650 700
Elevation [a.s.l.]
Profile metre [m]
Polarisation effect:
Q4
P3
P2
R1
P1
ORE
Phase -ϕ [mrad]
0 1 2 3 4 5 6 7 8 9 10
Elevation [a.s.l.]
Profile metre [m]
Legend Interpretation:
Late Bronze Age Dump
Lower egde mining areas
Debris - Limestone
Border Limestone
Border Porphyrite
