# Supplementary material for: Combining geophysical prospection and core drilling: Reconstruction of a Late Bronze Age copper mine at Prigglitz‐Gasteil in the Eastern Alps (Austria)
Source: Archaeol Prospect. 2022 Aug 2;29(4):557–77. doi: 10.1002/arp.1872 (PMC10087026; doi:10.1002/arp.1872)
Supplement: Supplementary file 5 — Figure S5. Prigglitz‐Gasteil. Profile P3: A resistivity, B induced polarization [file ARP-29-557-s002.pdf]

Electrical resistivity:

P3

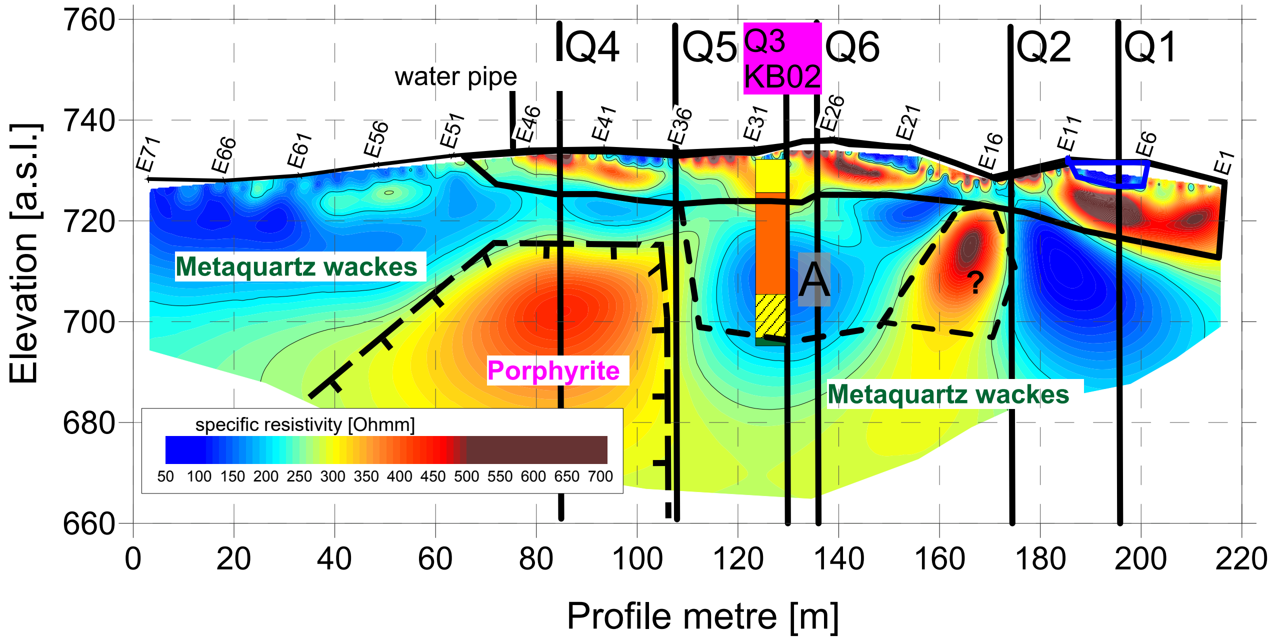

Polarisation effect:

P3

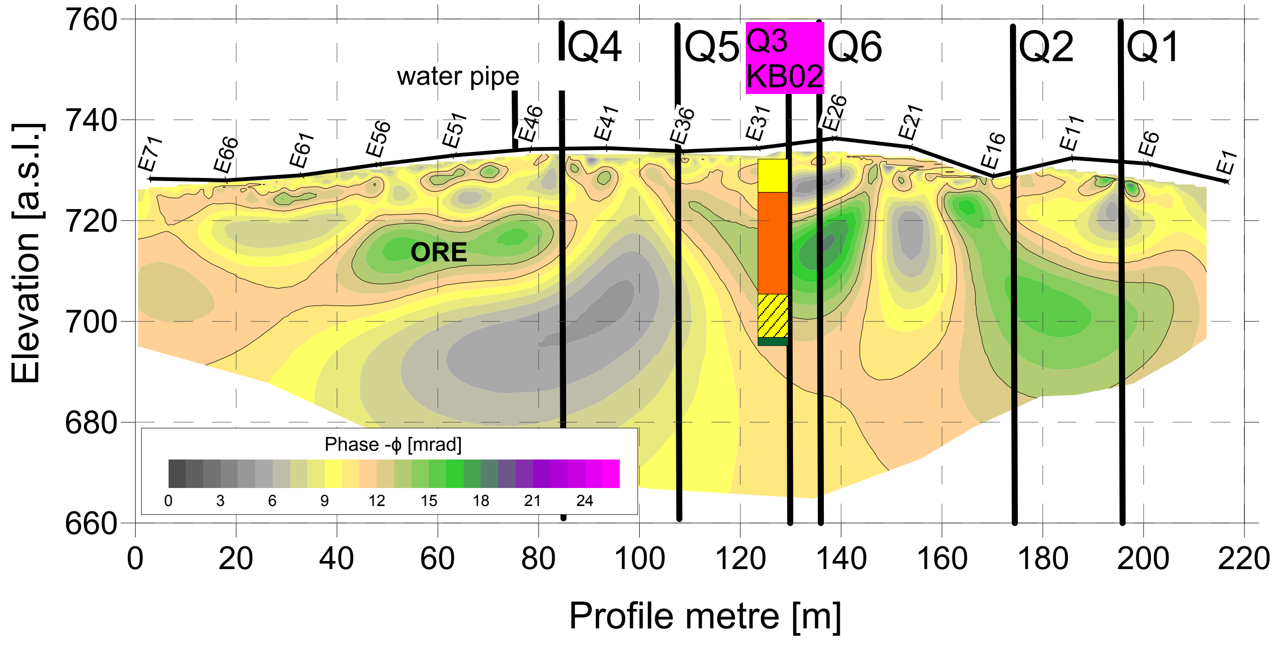

Legend Drilling:

- Upper mining Debris
- Landslip
- Lower mining Debris
- Bedrock

Legend Interpretation:

- Late Bronze Age Dump
- Lower egde mining areas
- Debris - Limestone
- Border Limestone
- Border Porphyite
