# Supplementary material for: Combining geophysical prospection and core drilling: Reconstruction of a Late Bronze Age copper mine at Prigglitz‐Gasteil in the Eastern Alps (Austria)
Source: Archaeol Prospect. 2022 Aug 2;29(4):557–77. doi: 10.1002/arp.1872 (PMC10087026; doi:10.1002/arp.1872)
Supplement: Supplementary file 7 — Figure S7. Prigglitz‐Gasteil. Profile P1: A resistivity, B induced polarization imaging results [file ARP-29-557-s001.pdf]

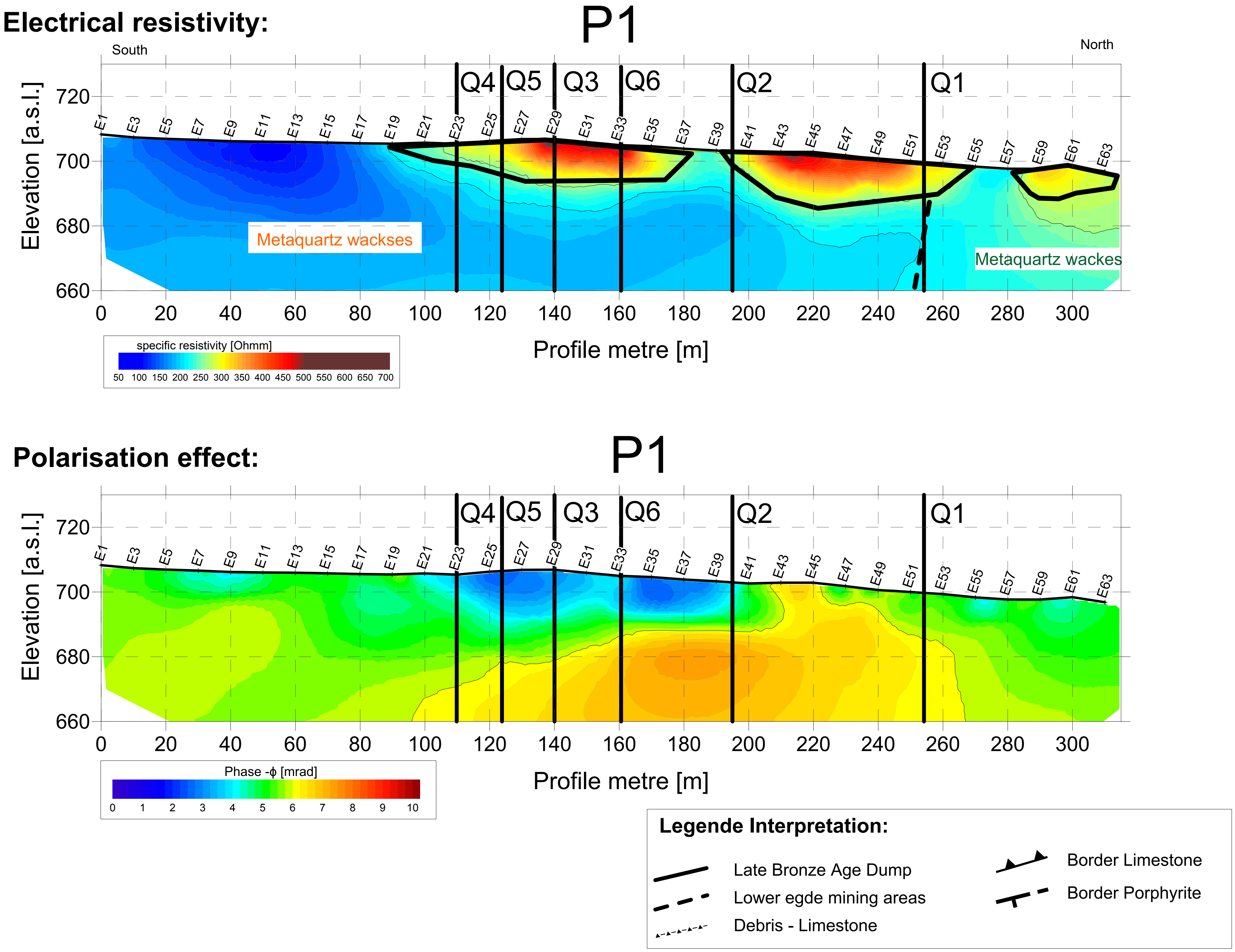

Electrical resistivity:
P1
South
North
Q4
Q5
Q3
Q6
Q2
Q1
Elevation [a.s.l.]
720
700
680
660
E1 E3 E5 E7 E9 E11 E13 E15 E17 E19 E21 E23 E25 E27 E29 E31 E33 E35 E37 E39 E41 E43 E45 E47 E49 E51 E53 E55 E57 E59 E61 E63
Metaquartz wackses
Metaquartz wackes
0 20 40 60 80 100 120 140 160 180 200 220 240 260 280 300
Profile metre [m]
specific resistivity [Ohmm]
50 100 150 200 250 300 350 400 450 500 550 600 650 700
Polarisation effect:
P1
Q4
Q5
Q3
Q6
Q2
Q1
Elevation [a.s.l.]
720
700
680
660
E1 E3 E5 E7 E9 E11 E13 E15 E17 E19 E21 E23 E25 E27 E29 E31 E33 E35 E37 E39 E41 E43 E45 E47 E49 E51 E53 E55 E57 E59 E61 E63
0 20 40 60 80 100 120 140 160 180 200 220 240 260 280 300
Profile metre [m]
Phase -ϕ [mrad]
0 1 2 3 4 5 6 7 8 9 10
Legende Interpretation:
Late Bronze Age Dump
Lower egde mining areas
Debris - Limestone
Border Limestone
Border Porphyrite
